# A Review of the Status of Brain Structure Research in Transsexualism

**DOI:** 10.1007/s10508-016-0768-5

**Published:** 2016-06-02

**Authors:** Antonio Guillamon, Carme Junque, Esther Gómez-Gil

**Affiliations:** 1 Departamento de Psicobiología, Universidad Nacional de Educación a Distancia, c/Juand del Rosal, 10, 28040 Madrid, Spain; 2 Academia de Psicología de España, Madrid, Spain; 3 Departamento de Psiquiatría y Psicobiología Clínica, Universidad de Barcelona, Barcelona, Spain; 4 Institute of Biomedical Research August Pi i Sunyer, Barcelona, Spain; 5 Unidad de Identidad de Género, Hospital Clinic, Barcelona, Spain

**Keywords:** Transsexualism, Sex differences, Gender identity, Gender dysphoria, Cross-sex hormone treatment, Magnetic resonance imaging

## Abstract

The present review focuses on the brain structure of male-to-female (MtF) and female-to-male (FtM) homosexual transsexuals before and after cross-sex hormone treatment as shown by in vivo neuroimaging techniques. Cortical thickness and diffusion tensor imaging studies suggest that the brain of MtFs presents complex mixtures of masculine, feminine, and demasculinized regions, while FtMs show feminine, masculine, and defeminized regions. Consequently, the specific brain phenotypes proposed for MtFs and FtMs differ from those of both heterosexual males and females. These phenotypes have theoretical implications for brain intersexuality, asymmetry, and body perception in transsexuals as well as for Blanchard’s hypothesis on sexual orientation in homosexual MtFs. Falling within the aegis of the neurohormonal theory of sex differences, we hypothesize that cortical differences between homosexual MtFs and FtMs and male and female controls are due to differently timed cortical thinning in different regions for each group. Cross-sex hormone studies have reported marked effects of the treatment on MtF and FtM brains. Their results are used to discuss the early postmortem histological studies of the MtF brain.

## Introduction

Transsexuals seek or have undergone a social transition from male to female (MtF) or female to male (FtM), a transition that in many, but not all, cases also involves a somatic transition by cross-sex hormone treatment and genital surgery (American Psychiatric Association, 2013; Meyer-Bahlburg, 2010, 2013).

Although the etiology of transsexualism is unknown, biological and environmental factors have been suggested to contribute to gender identity variations (Cohen-Kettenis & Gooren, 1999; Savic, Garcia-Falgueras, & Swaab, 2010; Lawrence & Zucker, 2014). Biological causes for gender dysphoria (GD) are supported by studies on familial groups (Gomez-Gil et al., 2010; Green, 2000), birth order (Blanchard & Sheridan, 1992; Blanchard, Zucker, Cohen-Kettenis, Gooren, & Bailey, 1996; Gomez-Gil et al., 2011; VanderLaan, Blanchard, Wood, Garzon, & Zucker, 2015; Vasey & VanderLaan, 2007), and twins (McKee, Roback, & Hollender, 1976; Zucker & Bradley, 1995). A review of the literature of twins concordant and discordant for GD suggests a role for genetics in the development of GD (Heylens et al., 2012). Molecular genetics have been used to analyze peripheral sex steroid-related polymorphisms in steroid receptors or steroid enzyme genes (Fernandez et al., 2014a, 2014b; Hare et al., 2009; Henningsson et al., 2005; Ujike et al., 2009). Research on prenatal androgen exposure markers has provided some evidence of transsexual differences based on the 2D:4D ratio (Schneider, Pickel & Stalla, 2006; Wallien, Zucker, Steensma & Cohen-Kettenis, 2008). The findings from all the above studies suggest that genetic factors could influence brain and behavioral phenotypes.

In regard to environmental variables, parental and family factors have been reviewed (Lawrence & Zucker, 2014); parental influences seem to be a contributing factor to the development of GID (Cohen-Kettenis & Gooren, 1999) and play a role in social gender transitioning (Steensma, McGuire, Kreukels, Beekman, & Cohen-Kettenis, 2013).

With respect to the developmental course of GD and sexual orientation, DSM-5 indicates that in both natally male and female children showing persistence, almost all are sexually attracted to individuals of their natal sex. Moreover, there are two broad trajectories for the development of GD: early-onset and late-onset. Early-onset GD starts in childhood and continues into adolescence and adulthood, while late-onset GD begins around puberty or even much later in life. Adolescent and adult natal males with early onset of GD are almost always androphilic, while most with a late onset are gynephilic. In natal females, the most common course is early-onset GD; they are almost always gynephilic, while the few with late-onset GD are usually androphilic (APA, 2013, pp. 455–456). Although DSM-5 criteria no longer include diagnostic subtyping by sexual orientation, early and late GD onset and sexual orientation have been stressed by those authors who distinguish two subtypes of MtFs and FtMs (Blanchard, 1989a, 1989b; Smith, van Goozen, Kuiper, & Cohen-Kettenis, 2005). Blanchard, taking into account the sex chromosomes at birth, has named androphilic MtFs homosexual and gynephilic MtFs nonhomosexual (Blanchard, 1989a, 1989b). However, Gooren had reservations about the use of the terms “homo”- and “nonhomosexual” because MtFs do not view themselves as homosexuals, considering themselves women in their sexual interaction with men (Gooren, 2006). The fact that two subtypes of MtFs and FtMs can be distinguished has important theoretical and clinical implications for the etiology of transsexualism (Blanchard, 2005). Consequently, distinctions between early- and late-onset GD and androphilic and gynephilic sexual orientation become essential when approaching the brain of transsexuals. Moreover, predictions for brain differences between MtF subtypes have been advanced in light of this distinction (Blanchard, 2008).

Brain sex differences have been used to study transsexuality. The approach was based on previous reports regarding the existence of morphological sex differences in the mammalian brain. It seems logical to compare the brains of MtFs and FtMs with brains from both male and female controls.

The early brain studies on transsexuality, directed toward the hypothalamus and the extended amygdala in postmortem human specimens, reported that the central part of the bed nucleus of the stria terminalis (BSTc) was feminine in MtFs (Zhou, Hofman, Gooren, & Swaab, 1995). More recently, a few groups have explored how brain sex differences are expressed in vivo in the brain of MtFs and FtMs using neuroimaging techniques.

This review focuses on the brain structure of early-onset GD androphilic (homosexual) MtFs and early-onset GD gynephilic (homosexual) FtMs. The early onset of GD and sexual orientation are key points in the following analysis. Our main aims are to (1) address the structural phenotype of the brain in homosexual MtFs and FtMs before cross-sex hormone treatment; (2) discuss these brain phenotypes in the light of the neurohormonal theory of sexual differentiation of the brain; (3) describe the effects of cross-sex hormone treatment on the structure of the brain; and (4) analyze the histological postmortem studies in light of the in vivo neuroimaging results. Investigating these objectives has suggested an explanatory hypothesis on gender. In approaching these objectives, we encountered several difficulties. The main one is the scant number of published MRI studies on the brain of transsexuals; this scarcity is more extreme in regard to nonhomosexual MtFs and FtMs. Moreover, some studies do not report sexual orientation or mix homosexual and nonhomosexual subjects.

## Morphological Characteristics of Sex Differences in the Mammalian Brain

### Neurohormonal Theory of Brain Sexual Differentiation

Observations arising from embryological and behavioral studies have guided research into the function of gonadal steroids in differentiating the brains of males and females at the morphological, physiological, and behavioral levels. These seminal works have shaped what is known as the neurohormonal theory of brain sexual differentiation, which is a key point to understanding the brain in relation to gender.

Jost (1947), working with gonadally indifferent rabbit embryos, showed that an indifferent urogenital tract goes through male differentiation if a testis develops and female differentiation if an ovary develops. Embryos gonadectomized before the indifferent gonad differentiates develop as phenotypically female. These experiments, carried out in the middle of the last century, demonstrate that the induced phenotype is male in mammals and that testicular secretions are necessary for further male development (for review, see Jost, 1972).

In the laboratory of Young (Phoenix, Goy, Gerall, & Young, 1959), a decade after Jost’s findings, a series of studies began on the sexual behavior of male and female guinea pigs born of mothers treated with testosterone propionate during pregnancy. Androgenized female pups were less likely to show lordosis and more likely to display mounting behavior in adulthood than were control animals when both were gonadectomized and treated with the appropriate sex hormones. It was suggested that testosterone administered prenatally had an organizing effect on the neural tissues mediating sexual behavior, while gonadal hormones activated these tissues and behavior when administered in adulthood. This hypothesis has guided research on the sexual differentiation of brain and behavior. However, there are behavioral traits that only require organizing actions by androgens, and no activational influence is necessary for their full expression by the individual; examples are juvenile play and mounting behavior in rhesus monkeys and the micturitional patterns of dogs (Goy & McEwen, 1980).

The organizational–activational hypothesis is the foundation for a unified theory of sexual differentiation of all mammal tissues (Arnold, 2009). However, some sex differences are not explained by gonadal hormonal effects, but by a primary action of genes encoded in the sex chromosomes. This understanding allowed Arnold to integrate the sex chromosome effects in the neurohormonal theory of brain and behavior differentiation.

The conceptual terminology was revisited 10 years ago by Becker et al. (2005). The words “masculine” and “feminine” are used to describe brain morphological or behavioral traits that are typical of the males or females of a species, respectively. Masculinization and feminization refer to any change that makes an individual more like typical males or females. Demasculinization and defeminization denote any change that makes an individual less like a typical male or female.

### Animal Studies

**Fig. 1:**
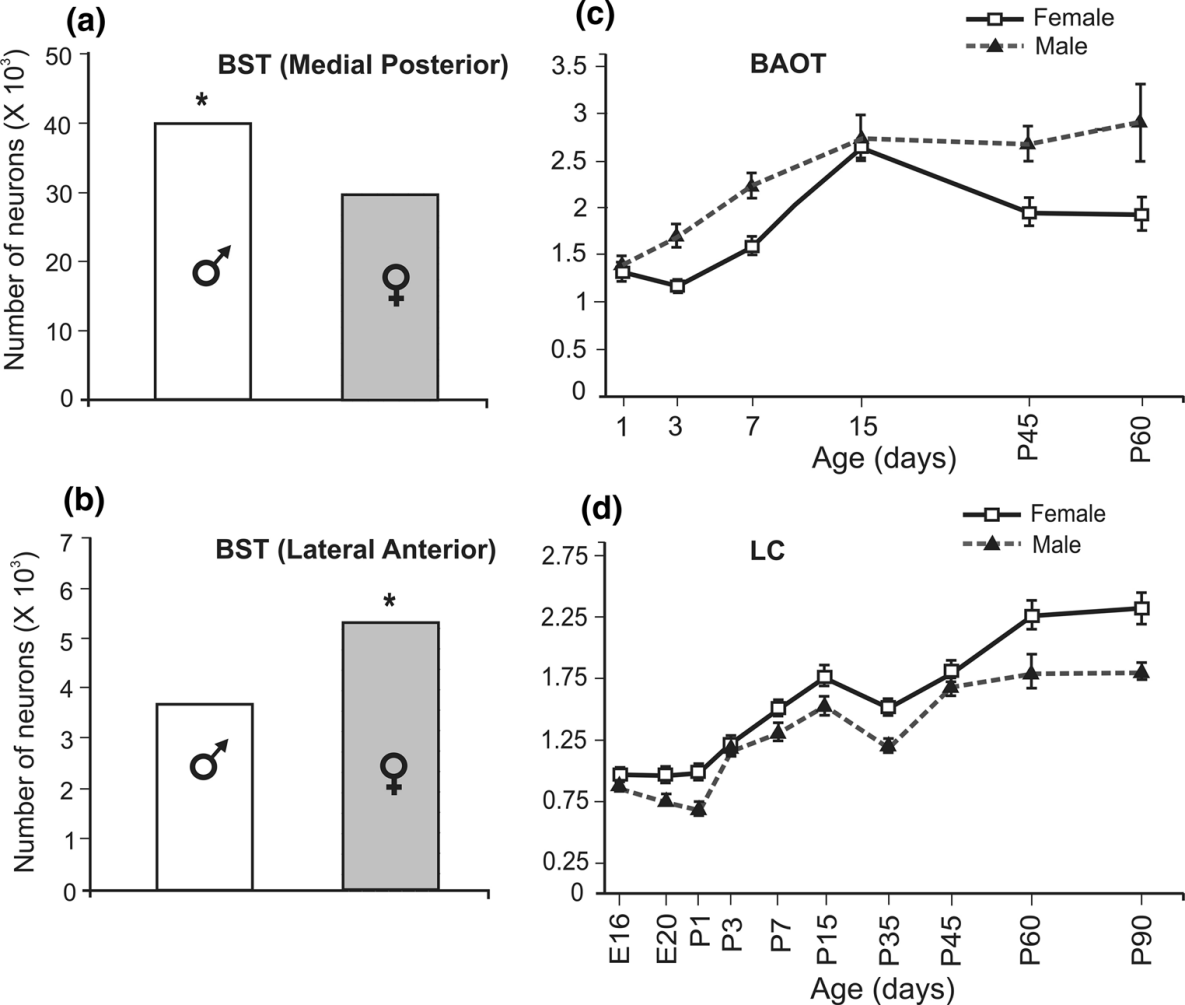
Main morphological characteristics of sex differences in the brain and their ontogeny. Sex differences in the brain present two morphological patterns as exemplified in the bed nucleus of the stria terminalis of the rat (BST). The medial posterior region of the BST has an M > F pattern, with males showing a greater number of neurons than females (**a**), while the lateral anterior region of the BST has an F > M pattern, with females showing more neurons than males (**b**). These two patterns of sex difference differ in their ontogeny as can be observed with respect to the number of neurons in the bed nucleus of the accessory tract (BAOT) (**c**) and the locus coeruleus (LC) (**d**) of rats. Puberty in the rat occurs between days 35 and 40. Both figures (**c**, **d**) show how females present decreases (**c**) and increases (**d**) in the number of neurons around this period of life in different structures. Figures adapted with permission from Guillamon, Segovia & Del Abril, 1988; Collado, Segovia & Guillamon, 1998 and Pinos, Collado, Rodriguez-Zafra, Rodriguez, Segovia & Guillamon, 2001. *E* embryonic days, *P* postnatal days

Studies on brain sex differences show three main morphological characteristics. The first is comparative size. Sex differences take one of two opposite morphological patterns in the adult brain (Segovia & Guillamon, 1993). Neurohistological studies reveal that in some brain structures males show greater morphological measurements (i.e., volume, number of neurons, dendrite spines, etc.) than females, while in other structures the opposite is true (Guillamon & Segovia, 1996; Segovia & Guillamon, 1993). Thus, the brain of each sex shows M > F and F > M morphological patterns, according to the region studied (Fig. 1a, b). Of course, there are brain structures that, comparing sexes, are isomorphic (M = F). Morphologically, the cerebral pattern of M > F, F > M, and M = F structures determines the sex of the brain. Below, we will see that male and female patterns reflect different growth programs for particular structures. The fact that brain sex differences are present in two opposite morphological patterns (M > F and F > M) is a key point to understanding (1) what is truly feminine or masculine in the brain and (2) how the concepts of masculinization, demasculinization, feminization, and defeminization in brain morphology can be correctly applied in a given sex. If a structure has an F > M pattern of sex differences (to be masculine in this pattern is to have smaller morphological measurements than females), then feminization of this structure in males would mean an increase in the morphological measurements resulting in an M = F pattern for that structure, while demasculinization would mean a variation of the morphological parameters making that structure significantly different from both F and M.

The second characteristic is that brain sex differences are present in complex networks involving many regions (Cosgrove, Mazure, & Staley, 2007; Segovia & Guillamon, 1993; Simerly, 2002). As an example, the accessory olfactory system, implicated in the control of sexual and maternal behaviors, is known to be sexually dimorphic in rodents (Segovia & Guillamon, 1993) and lagomorphs (Segovia et al., 2006). The olfactory system also shows sex differences in humans (Garcia-Falgueras et al., 2006).

Third, animal ontogenetic studies of the M > F and F > M patterns show two important aspects as to how sex differences are built up in the brain. Natural cell death (apoptosis) and neurogenesis are intrinsic to sex differences’ development and are differently timed in different regions.

The bed nucleus of the accessory olfactory tract (BAOT) in the rat, which belongs to the accessory olfactory system network (de Olmos, Hardy, & Heimer, 1978), is sexually dimorphic and presents an M > F pattern with respect to its volume and number of neurons (Collado, Guillamon, Valencia, & Segovia, 1990). Sex differences in this nucleus are controlled in the early postnatal days by testosterone, probably aromatized to estradiol (Collado et al., 1990) and, as shown in Fig. 1c, these sex differences are already present very early in development; puberty may just enhance them further through the decrease in neuron number that occurs in females at that time (Collado, Segovia, & Guillamon, 1998). This suggests that sex differences in the M > F pattern are due to apoptosis occurring around puberty in the female.

The locus coeruleus, the main origin of noradrenergic projections to the brain, presents an F > M pattern of sex differences in rats with respect to its volume and neuron numbers (Guillamon, de Blas, & Segovia, 1988). Ontogenetic studies from embryonic days to adulthood show that, in females, the number of neurons in this nucleus increases until adulthood, while, in males, it plateaus at day 45 after birth (Fig. 1d; Pinos et al., 2001). Puberty, which occurs between days 35 and 40 in the rat, seems to be important for building up sex differences.

There are a substantial number of behavioral works with animals showing that gonadal hormones secreted during puberty initiate a second period of brain organization in males and females (Schulz, Molenda-Figueira, & Sisk, 2009). New cells, including neurons, arise in M > F and F > M brain regions during puberty and removing the gonads before puberty eliminates this mechanism of sexual differentiation (Ahmed et al., 2008).

### Human Studies

Sex differences in the human brain have the same morphological characteristics observed in animal studies. Postmortem anatomical (Dekaban & Sadowsky, 1978; Pakkenberg & Gundersen, 1997; Rabinowicz, Dean, Petetot, & de Courten-Myers, 1999) and in vivo MRI studies (Cosgrove et al., 2007; Luders & Toga, 2010; Ruigrok et al., 2014) consistently report 9–12 % greater intracranial volume (ICV) in adult males than females as well as in children (Lenroot & Giedd, 2010). A recent MRI meta-analysis confirms that ICV shows robust sex differences and suggests a bias towards 18- to 59-year-olds with respect to ICV, white matter (WM), gray matter (GM), and cerebrospinal fluid (CSF) volumes (Ruigrok et al., 2014). The pattern for all these parameters is M > F (Table 1).

**Table 1:** Sex differences in intracranial volume and brain compartments of the adult brain

Brain region/aspect	Sample size and age (*M* ± *SD* or range)	Sample source	Brain-relevant sample characteristics	Brain imaging methods and measurements	Statistically controlled confounding variables	Selected findings and conclusions	Pattern	Authors
ICV GM WM CSF	40 M; 40 F Age: 18–45 (whole sample)	Recruited by advertisement Department Psychiatry, University Pennsylvania (USA)	Healthy Right-handed Ss F: premenopausal	1.5 T General Electric scanner Brain volume extracted automatically MRI segmentation GM, WM, CSF *Measurements* Volume; % respect ICV; cortical surface	Age ICV	Volume, M > F: ICV, GM, WM, CSF When adjusted by ICV:	All volumes and % M > F, except for % GM: F > M	Gur et al. (1999)
						% GM: F > M		
						% WM: M > F		
						% CSF: M > F		
						*Conclusions* SD depending on brain compartment		
ICV GM WM	42 M; 42 F Age: M:19–31 F: 19.31	Recruited by advertisement College of Medicine, University of Iowa (USA)	Healthy volunteers matched for age, education, IQ	1.5 T General Electric Signa scanner Processing imaging by locally developed software *Measurements* Volume; % respect ICV; surface anatomy	Height as covariate for all measurements	ICV: M > F When adjusted by ICV: % GM F > M in both parietal lobes *Conclusions* SD depends on cerebral lobes	M > F: ICV F > M: % GM parietal lobes	Nopoulos et al. (2000)
ICV GM WM	22 M; 32 F Range: 20–86 M: 44.7 ± 19.9 F: 48.3 ± 19.9	Fox Chase Cancer Center, Philadelphia (USA)	Healthy volunteers no differences in age between M and F	1.5 T General Electric Signa scanner Volumes segmented by semi automated software *Measurements* volume	Age	M > F: ICV *Conclusions* SD in ICV not in % GM controlled by ICV.	M > F: ICV	Ge et al. (2002)
ICV GM WM CSF	Sample matched by ICV: 24 M, 24 F Age range: M: 21–61; F: 19–69 Extreme sample (F with the smallest ICV and M with the largest ICV) 24 M; 24 F Age range: M: 18–69; F: 19–65	ICBM data base for normal adults. USA, Germany, Canada. Brain images from USA	M and F with similar brain size	1.5 T Siemens Sonata VBM Corrected (FDR) *Measurements* regional volumes	Age ICV	No significant interaction of ICV × Sex All Ss (48 M vs. 48 F) ICV: M > F Matched by ICV (24 M and 24 F): No differences in ICV, GM, WM, and CSF ratios. Regional GM In regions of the four lobes and caudate nucleus: Matched F> Matched M. *Conclusions* Anatomical differences between M and F exist independently of brain size effects	M > F: ICV F > M: GM in regions of four lobes	Luders, Gaser, Narr, and Toga (2009a)

*ICV* intracraneal volume, *GM* gray matter, *WM* white matter, *CSF* cerebrospinal fluid (includes ventricles and cisterns), *FDR* false discovery rate, *VBM* voxel-based morphometry, *F* female, *M* male, *Ss* subjects, *SD* sex differences, *ICBM* International Consortium for Brain Mapping

However, when regional aspects of the brain are approached and ICV controlled, the F > M pattern of volumetric sex differences is observed. The pattern F > M emerges in the percentage of GM and the volume of the cortex. Subcortical regions and the percentage of WM show an M > F pattern (Tables 1, 2).

**Table 2:** Sex differences in cortical and subcortical structures of the adult brain

Brain region/aspect	Sample size and age (*M* ± *SD* or range)	Sample source	Brain-relevant sample characteristics	Brain imaging methods and measurements		Statistically controlled confounding variables	Selected findings and Conclusions	Pattern	Authors
Cortex	30 M; 30 F M: 25.45 ± 4.72 F: 24.32 ± 4.35	Images from Center for Scientific Innovation and Technology, Magdeburg, Germany	Healthy young M and F	1.5 T General Electric Loni software *Measurements* CTh		ICV Age	Without ICV correction, F > M in the four lobes With ICV correction, still F > M, except for a small region in the temporal lobe *Conclusions* F have thicker cortex than M	F > M	Luders et al. (2006)
Cortex	90 M; 94 F Age range: M: 18–67 F: 18–70	Normal Ss from TongRen Hospital, Beijing, China	Healthy adult M and F, population from Asia	1.5 General Electric Signa scanner CTh measured using an automated surface method + graph theoretical approaches *Measurements* CTh		ICV Age	F > M: Frontal, parietal and occipital lobes. M > F: Small portions of temporal lobes *Conclusions* F thicker cortex than M. In small regions M > F	F > M M > F (small regions)	Lv et al. (2010)
Cortex	31 M; 21 F Age range: M: 18–42 F: 19–36	Seoul Normal Ss from National University Hospital, Seoul, Korea	Healthy adult M and F Population from Asia	1.5 T General Electric Signa scanner *Measurements:* CTh		ICV Age	F > M: frontal, parietal and occipital lobes Temporal lobes show relatively less significant thickening in F *Conclusions* F thicker cortex than M	F > M	Im et al. (2006)
Cortex	90 M; 86 F M: 31 ± 21.3 F: 33.9 ± 22.3 Age range whole sample: 7–87	Normal Ss community sample, Department of Psychiatry, Columbia University, New York, USA	A large age range population	1.5 T General Electric Signa GM thickness calculated using Eikonal fire equation *Measurements* CTh		Age ICV body size	The thicker cortices in F than M: right inferior parietal and posterior temporal independent of differences in body size and ICV *Conclusions* F thicker cortex than M	F > M	Sowell et al. (2007)
Cortex, amygdala and hypothalamus	27 M; 21 F M: 39.13 ± 12 F:36.3 ± 10.5	Normal, 93% Caucasian Ss. Department of Psychiatry, Harvard Medical School, Boston, USA	Healthy adults, M and F same ethnicity, education	1.5 T General Electric Signa Semiautomatic delimitations *Measurements* Volume		Age ICV	Volume: M > F amygdala and hypothalamus F > M: cortex *Conclusions* M greater volumes in amygdala & hypothalamus	M > F F > M	Goldstein et al. (2001)
Amygdala and hippocampus	313 M; 306 F Age range for the whole sample 40–90	Mental Health Institute of Beijing Medical University, China	Large population of normal adults from Asia	1.5 T MR unit Volume of structures Manually outlined using as a brain atlas slices from 2 postmortem specimens *Measurements* Volume			Volume of amygdale and hippocampus declines with age No sex differences *Conclusions* No SD in large sample no caucasian	M = F	Mu et al. (1999)
Amygdala and hippocampus	57 M; 59 F M:27.0 **±** 5.7 F:25.0 **±** 5.3	Volunteers Department of Psychiatry, University Medical Center, Philadelphia, USA	Healthy adult population	1.5 T General Electric Signa Brain volumes extracted automatically ‘ROIS manually outlined for subcortical structures *Measurements* Volume		ICV	M and F have similar volume in amygdale and hippocampus *Conclusions* No SD	M = F	Gur et al. (2002)
Basal ganglia	463 M; 541 F Age range whole sample: 18–36	Ss enrolled in the Brain Imaging Genetic project at Medical Center, Radboud University, Nijmegen, Netherlands	Large healthy population	1.5 and 3 T scanners Automatic volumetry on MRI images *Measurements* volume		Age Separate cohorts for the two type of scanners Control for total GM and WM	M > F putamen and globus pallidus No SD for caudate nucleus and nucleus accumbens *Conclusions* some basal ganglia show SD	M > F	Rijpkema et al. (2012)
Cortex and subcortical structures	40 M; 51 F Age range whole sample: 18–33	Healthy young adults. Department of Psychiatry and clinical Psychobiology, University of Barcelona, Spain	Healthy adult population	1.5 T General Electric scanner VBM ROIs analyses of olfactory system structures Uncorrected *Measurements* concentration		Age	M > F: BA 28, pallidum. F > M: BA10, 11, 25, hippocampus, amygdala *Conclusions* subcortical regions show both M > F and F > M patterns of SD	M > F F > M	García-Falgueras et al. (2006)
Temporal lobe Superior temporal gyrus Amygdala Hippocampus	53 M, 46 F Age range all Ss: 4.7–17.8	Recruited from the community, Child Psychiatric Branch, National Institute of Mental Health, Bethesda, USA	Healthy young population	1.5 T Signa Advance scanner Manual tracing *Measurements* volume		Age Handedness Tanner stage ICV	R Amygdala increases only in M, while R Hippocampus increases only in F *Conclusions* SD in maturational changes	M > F F > M	Giedd et al. (1996)

*ICV* intracraneal volume, *GM* gray matter, *WM* white matter, *CTh* cortical thickness, *BA* Brodmann’s area, *VBM* voxel-based morphometry, *SD* sex differences, *F* female, *M* male, *Ss* subjects

Brain cortical thickness (CTh) studies measure the distance between the pia mater and white matter in tens of thousands of points in the cortex and provide better information than those using voxel-based morphometry (VBM) because cortical volume measurements obtained by VBM procedures blend the effects of cortical surface and folding (Panizzon et al., 2009; Winkler et al., 2010). CTh studies have shown that females have a thicker cortex than males, even in studies that control for ICV, body size, and age (Table 2).

The best technique to study the microstructure of the WM is Diffusion Tensor Imaging (DTI). It measures diffusivity of the water molecules within the axons and detects subtle changes in the WM and has been used in developmental (Huster, Westerhausen, Kreuder, Schweiger, & Wittling, 2009; Schmithorst, Holland, & Dardzinski, 2008; Westerhausen et al., 2003) and psychiatric studies (Nucifora, Verma, Lee, & Melhem, 2007). DTI measures fractional anisotropy (FA), which indicates white matter coherence and axonal organization (Lebel, Walker, Leemans, Phillips, & Beaulieu, 2008). Another parameter used to assess white matter integrity is mean diffusivity (MD) values—they are complementary information to FA with high MD values indicating loss of white matter integrity, while a low FA reflects the same (Lebel et al., 2008). As shown in Table 3, there are sex differences in the WM microstructure and, depending on the region, they present an M > F pattern.

**Table 3:** Sex differences in white matter microstructure of the adult brain

Brain region/aspect	Sample size and age (M ± SD or range)	Sample source	Brain-relevant sample characteristics	Brain imaging methods and measurements	Statistically controlled confounding variables	Selected findings and conclusions	Pattern	Authors
White matter microstructure	Right-handed: 16 M; 18 F Left-handed: 16 M; 17 F Age range whole sample: 19–34	Ss: Center for Neuropsychol. Research, students from University of Trier, Germany	Healthy young adults, handedness	1.5 Philips Intera DTI *Measurements:* FA, MD in whole corpus callosum (CC)	Age Handedness	CC: L>R M > F *Conclusions* SD in microstructure CC	M > F	Westerhausen et al. (2003)
White matter microstructure	20 M; 13 F 8 B; 7G Range: M + F: 22–64 B + G:14–21	Ss: Volunteers Department Psychiatry Columbia University, New York, USA	Comparison healthy adults and adolescents	1.5 T General Electric DTI; ROIS of 13 regions *Measurements* FA	Age	CC: M > F and F > M F > M: L Cingulum SD in the majority of fascicles, most marked in Cingulum F > M in L Cingulum M > F R Cingulum *Conclusions* SD in relation to the hemisphere and region	M > F F > M	Schneiderman et al. (2007)
White matter microstructure	Right-handed: 17 M; 17 F Left-handed: 21 M; 24 F Age range whole sample: 19–34	Ss: Center for Neuropsychol. Research, students from University of Trier, Germany	Healthy young adults, handedness	1.5 Philips Intera DTI *Measurements* In cingulum: FA and MD WM volume	Handedness ICV	FA more discriminative than MD for gender *Conclusions* SD in microstructure of Cingulum	M > F	Huster et al. (2009)

*ICV* intracraneal volume, *GM* gray matter, *WM* white matter, *CC* corpus callosum, *VBM* voxel-based morphometry, *DTI* diffusion tensor imaging, *FA* fractional anisotropy, *MD* mean diffusivity, *SD* sex differences, *L* left, *R* right, *F* female, *M* male, *Ss* subjects, *B* boys, *G* girls

Structural MRI studies also show sex differences in age-related brain volume (Brain Development Cooperative Group, 2012; Sowell et al., 2007) and puberty seems to play a significant role in the developmental course of human white (Giedd et al., 1999; Perrin et al., 2008) and gray matter (Raznahan et al., 2010; Shaw et al., 2008; see also Tables 1, 2, 3).

Finally, using functional techniques, sex differences have been reported in the connectome, with males having greater intra-hemispheric connectivity, while, in females, inter-hemispheric connectivity predominates (Ingalhalikar et al., 2014).

## The Brain Phenotype of Untreated Transsexuals

### The Brain of Male-to-Female Homosexual Transsexuals Before Cross-Sex Hormone Treatment

#### Volume and Brain Compartments

MRI studies show that ICV in adult (Rametti et al., 2011b) and adolescent (Hoekzema et al., 2015) untreated homosexual MtFs is similar to male controls’. Moreover, GM, WM, and CSF volumes in homosexual MtFs do not differ from those of control males and are significantly greater than those of control females (Table 4).

**Table 4:** Brain volume of untreated homosexual male-to-female transsexuals

Brain region/aspect	Normative sex differences (M vs. F)	Sample size and age (*M* ± *SD*)	Sample source	Brain-relevant sample characteristics	Brain imaging methods and measurements	Statistically controlled confounding variables	Selected findings and conclusions	Authors
Whole brain GM WM CSF	M > F	18 untreated early-onset GD Hom MtF 19 Het F 19 Het M MtF: 24.71 ± 8.15 Het F:33 ± 8.23 Het M: 31.94 ± 6.11	Hospital Clinic, Barcelona, Spain	Untreated early-onset GD MtFs	3 T Siemens Trio SIENAX *Measurement* ICV	Age SD	MtF = M: ICV, GM, WM, CSF *Conclusions* In MtF ICV and GM, WM, and CSF compartments are masculine	Rametti et al. (2011b)
Whole brain	M > F	11 Untreated androphilic MtF adolescents: 44 M and 52 F MtFs: 13.77 ± 2.42 M: 16.42 ± 2.75 F: 16.29 ± 2.96	Center of Expertise on Gender Dysphoria ,VU University, Amsterdam, Netherlands	Untreated adolescent MtFs	VBM *Measurement* Intracranial volume	Tanner stage	MF>F *Conclusions* ICV masculine	Hoekzema et al. (2015)

*ICV* intracranial volume, *GM* gray matter, *WM* white matter, *CSF* cerebrospinal fluid (includes ventricles and cisterns), *FDR* false discovery rate, *VBM* voxel-based morphometry, *SD* sex differences, *M* male, *F* female, *Ss* subjects, *MtF* male-to-female transsexuals, *Hom* homosexual, *Het* heterosexual

#### Cortex

There are two volumetric studies of the cortex using voxel-based morphometry (VBM) in adolescent and adult untreated MtFs (Table 5). These studies compare MtFs with male and female controls that show sex differences. Simon et al. (2013) have studied a small sample of untreated homosexual MtFs. Homosexual MtFs and female controls had less gray matter volume in the left somatosensory and primary motor cortices as well as the posterior cingulate and calcarine gyri and the precuneus than male controls and FtMs. These findings suggest that homosexual MtFs have a feminine cortical pattern. However, the results should be taken cautiously because of the small sample size and the brain statistical maps showing significance were at an uncorrected level (*p* < .001).

**Table 5:** The cortex and the white matter of untreated homosexual male-to-female transsexuals

Brain region/aspect	Normative sex differences (M vs. F)	Sample size and age (*M* ± *SD* or range)	Sample source	Brain-relevant sample characteristics	Brain imaging methods and measurements	Statistically controlled confounding variables	Findings and conclusions	Authors
Cortex	F > M	10 Hom MtF 7 Hom FtM 7 M 11F MtFs: 228.8 ± 7.69 M: 27.1 ± 75.54 F: 23.9 ± 73.424	Psychiatry and Psychotherapy Department, Semmelweis University, Budapest, Hungary	Untreated Ho MtFs Sexual orientation	3 T Philips Achieva scanner VBM Cluster threshold: 30 voxels. Uncorrected *Measurements* Volume	Age	M > F:Right posterior cingulated; precuneus F > M: Right superior temporal gyrus F&MtF<M&FtM: Left pre-postcentral gyrus, posterior cingulated, calcarine gyrus, and precuneus F&MtF>FtM&M: Right occipital lobe (middle inferior occipital, fusiform, and lingual gyri. Right inferior temporal gyrus *Conclusions* Differences between transsexuals and control same biological gender	Simon et al. (2013)
Cortex	F > M	11 Untreated androphilic MtF adolescents 44 M adolescents 52 F adolescents MtFs: 13.77 ± 2.42 M: 16.42 ± 2.75 F: 16.29 ± 2.96	Center of Expertise on Gender Dysphoria VU University Medical Center, Amsterdam, Netherlands	Untreated adolescent androphilic MtF Sexual orientation	3 T Philips Intera scanner VBM c Clusters threshold >10 voxels FWE corrected *Measurements* Volume	Tanner stage	MtF<M: Left superior hemisphere of cerebellum. MtF<F: Right inferior orbitofrontal cortex *Conclusions* In MtF adolescents the volume of cortex differs from M and F controls in some regions	Hoekzema et al. (2015)
Cortex	F > M	18 Hom MtFs 24 Hom FtM 29 Het M 23 Het F Hom MtF: 25.50 ± 6.91 Hom FtM: 26.21 ± 9.50 Het M. 29.28 ± 6.35 Het F: 31.09 ± 8.64	Hospital Clinic, Barcelona, Spain	Early GD onset untreated young adults Ho MtF Sexual orientation	3 T TIM TRIO Siemens scanner CTh analysis by FreeSurfer FWE correction *Measurements* CTh	Age	F > M: Left inferior parietal; Right postcentral; Right pars triangularis. MtF> M: Right: rostral middle frontal; cuneus; medial orbitofrontal *Conclusions* MtFs thicker (feminine) CTh than M, but differed from M in regions that F do not (see Fig. 4)	Zubiaurre-Elorza et al. (2013)
White matter	M > F	18 Hom MtF 19 Het F 19 Het M MtF: 24.71 ± 8.15 Het F:33.00 ± 8.23 Het M: 31.94 ± 6.11	Hospital Clinic, Barcelona, Spain	Early GD onset untreated Ho MtF Sexual orientation	3 T TIM TRIO Siemens scanner *Measurements* FA	Age	MtFs<M and MtFs>F Right: superior longitudinal fasciculus. Cingulum Forceps minor Corticospinal tract Left: Superior longitudinal fasciculus *Conclusions* The main fascicles of the right hemisphere are demasculinized in MtFs	Rametti et al. (2011b)

*ICV* intracraneal volume, *GM* gray matter, *WM* white matter, *CSF* cerebrospinal fluid (includes ventricles and cisterns), *CTh* cortical thickness, *FA* fractional anisotropy, *M* male, *F* female, *Ss* subjects, *FDR* false discovery rate, *VBM* voxel-based morphometry, *FWE* family-wise error correction, *Het* heterosexual, *Hom* homosexual, *GD* gender dysphoria

More recently, the gray matter of untreated androphilic GD adolescents has been addressed (Hoekzema et al., 2015). MtFs have smaller volume than male controls in the left superior posterior hemisphere of the cerebellum and smaller volume than female controls in the right inferior orbitofrontal cortex. Thus, untreated MtF adolescents differ from both male and female controls in some cortical regions.

**Fig. 2:**
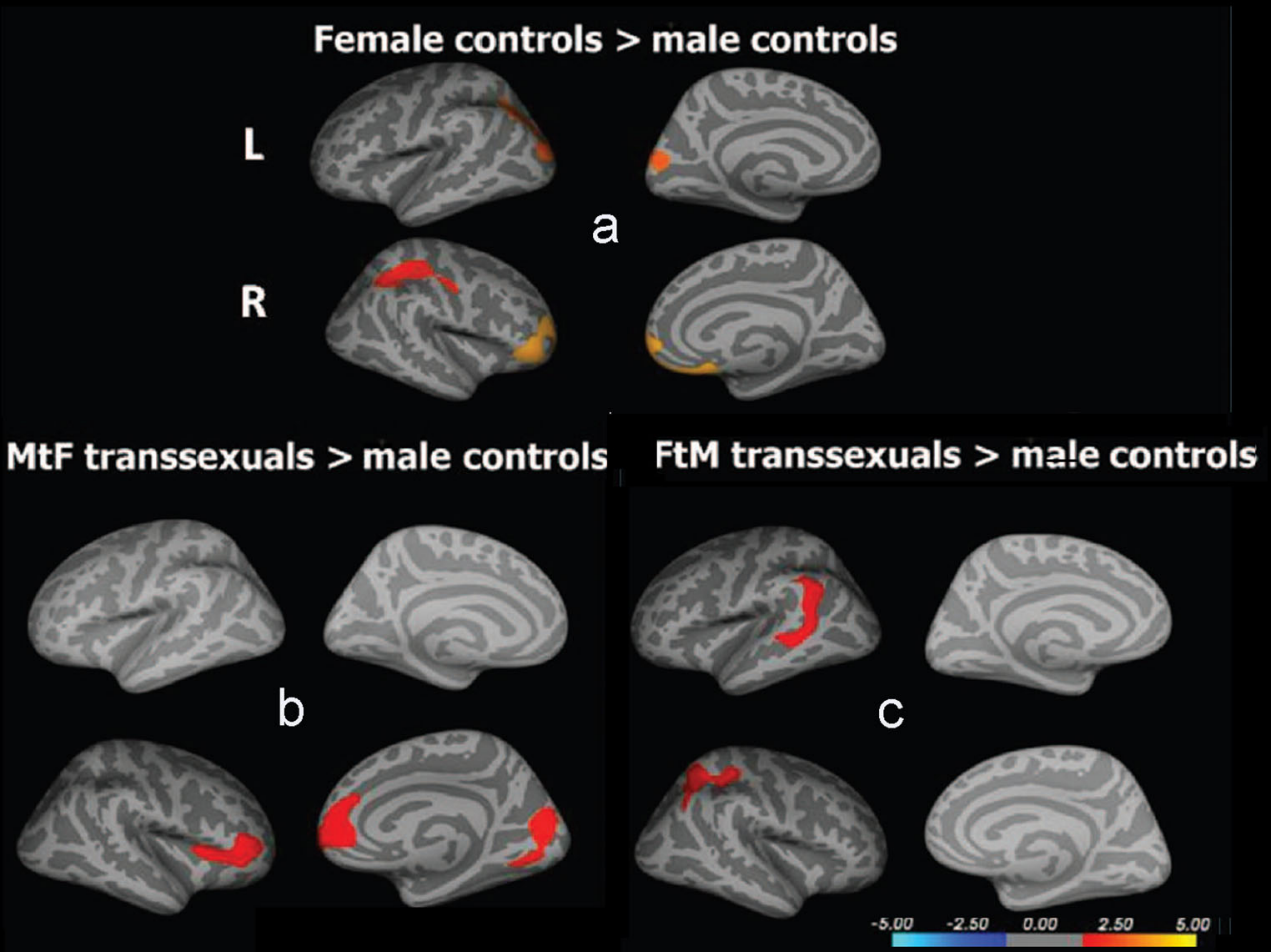
Cortical thickness of untreated homosexual male-to-female (MtF) and female-to-male (FtM) transsexuals. *Upper panel*: (**a**) comparison between male and female controls. *Bottom panel*: (**b**) comparison between MtF and male controls; **c** comparison between FtM and male controls. All significant comparisons showed the F > M pattern. Note that both MtFs (**b**) and FtMs (**c**) show a feminine pattern although they differ in different regions from males than do control females. *L* left hemisphere, *R* right hemisphere. Zubiaurre-Elorza, Junque, Gómez-Gil, Segovia, Carrillo & Guillamon, 2013, with permission

CTh has also been used to investigate brain differences in transsexuals (Table 5). Zubiarre-Elorza et al. (2013) compared early-onset untreated homosexual MtFs with female and male controls. MtFs did not differ in CTh from female controls but their CTh was greater than that of control males in the orbitofrontal, insular, and medial occipital regions of the right hemisphere. This report was the first to show the feminization of large portions of the cortex in early-onset homosexual MtFs and it concluded that MtFs had a feminine cortical thickness but differed from control males in regions that female controls did not (Table 5; Fig. 2b).

#### White matter

**Fig. 3:**
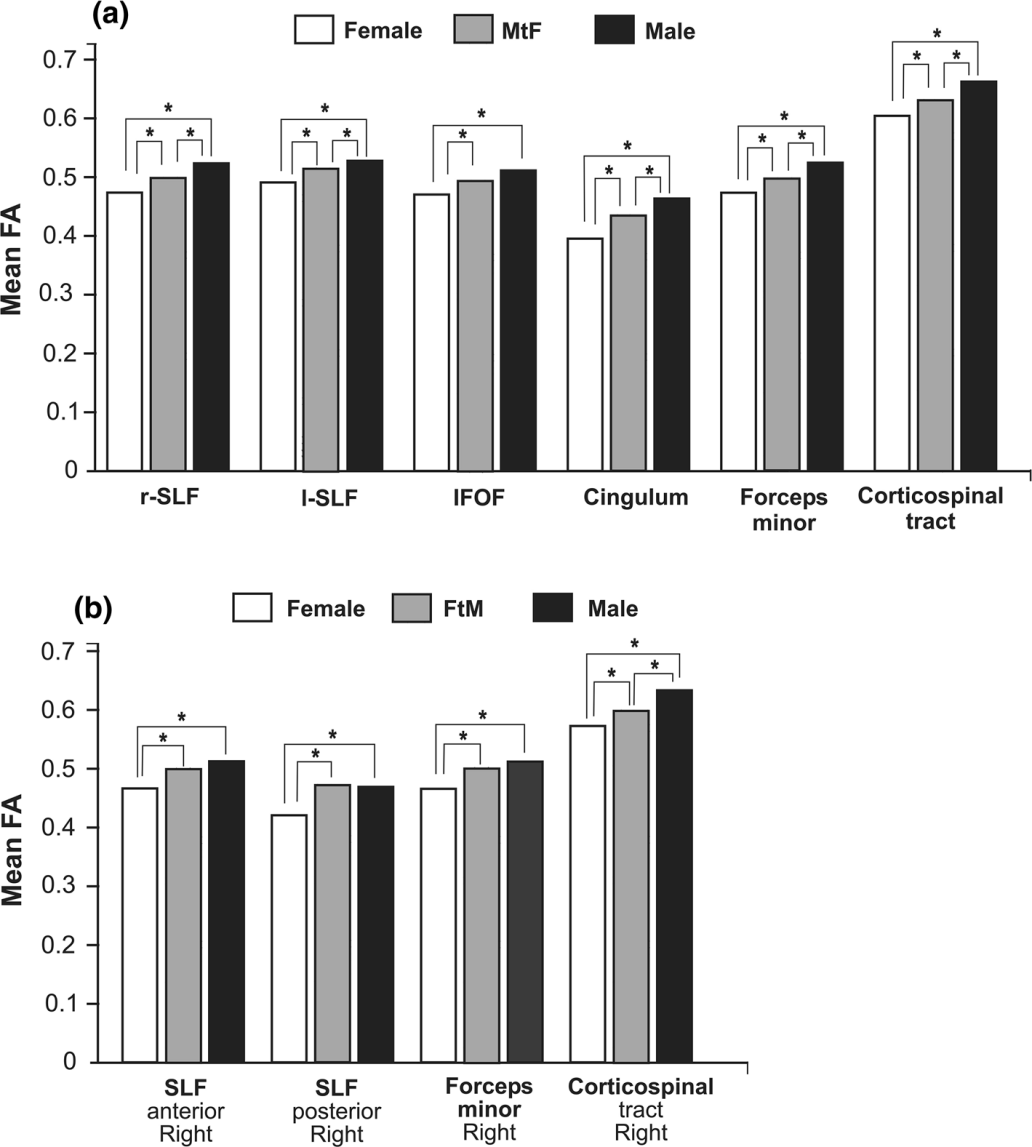
Histograms showing fractional anisotropy mean values (FA) of untreated homosexual male-to-female (MtF) and female-to-male (FtM) transsexuals and male (M) and female (F) controls. *Upper panel*: (**a**) FA values in MtF differ significantly from females in all six comparisons, and from males in only five out of the six. *Bottom panel*: (**b**) FA values in FtMs differ significantly from females in all four comparisons, from males in only one out of the four. *SLF* superior longitudinal fasciculus (*r* right, *l* left), *IFOF* inferior fronto-occipital fasciculus. Rametti et al. 2011a and 2011b, with permission

Recently, white matter microstructure has been studied in early-onset homosexual MtFs using DTI (Table 5; Fig. 3a). There are sex differences in FA, males showing greater FA values in important brain fascicles such as the right and left superior longitudinal fasciculi (rSLF; lSLF), the inferior fronto-occipital fasciculus (IFOF), the cingulum (Cin), the forceps minor (Fm), and the corticospinal tract (CST) (Rametti et al., 2011b). Interestingly, early-onset homosexual MtFs show demasculinized FA in all these brain fascicles because their FA values were statistically different from the values for both the male and female control groups. The MtF IFOF is masculine because its FA did not differ from male controls’. Curiously, the demasculinized fascicles seem to be restricted to the right hemisphere (Table 5; Rametti et al., 2011b).

#### Conclusions

Overall, in vivo MRI studies indicate that the main morphological parameters of the brain (ICV, GM, WM, and CSF) are congruent with their natal sex in untreated homosexual MtFs. However, some cortical regions show feminine volume and thickness and it should be underscored that CTh presents an F > M morphological pattern. Nevertheless, with respect to CTh, this feminine cortical pattern is not the same as the one shown by control females (compare Fig. 2a and b). On the other hand, the main white matter fascicles in MtFs are demasculinized, while others are still masculine (Fig. 3a). Moreover, most of the differences appear to be located in the right hemisphere. So far, the studies on the white matter, like those above on gray matter, strongly suggest that MtFs have their own brain phenotype that mainly affects the right hemisphere.

### The Brain of Nonhomosexual Male-to-Female Transsexuals Before Cross-Sex Hormone Treatment

All we know about the morphology of the brain of nonhomosexual MtFs comes from a single VBM study (Savic & Arver, 2011). Nonhomosexual MtFs have the same total intracranial volume as control males. They also show a larger gray matter volume in cortical regions in which the male and female controls did not differ in the study. These regions were the right parieto-temporal junction, the right inferior frontal, and the insular cortices. It was concluded that their data did not support the notion that the nonhomosexual MtF brain was feminized.

With respect to subcortical structures, it was reported that untreated nonhomosexual MtFs had a relatively smaller putamen and thalamus than male and female controls although these two latter groups did not show sex differences in the two structures (Savic & Arver, 2011).

In summary, the cortex of nonhomosexual MtFs presents morphological peculiarities in regions in which male and female controls do not differ.

### The Brain of Female-to-Male Transsexuals Before Cross-Sex Hormone Treatment

#### Brain Morphology

Although there are few works on the brain morphology of FtMs as yet, several studies have described the gray and white matter of untreated homosexual FtMs. These works can give us an initial approach to the morphology of their brain (Rametti et al., 2011a; Zubiaurre-Elorza et al., 2013). In regards to the gross morphology, the intracranial volume of adolescent FtMs is similar to female controls’ (Hoekzema et al., 2015).

There are only two works using VBM (Table 6). Simon et al. (2013) studied seven homosexual FtMs and found that these subjects and their male controls had larger volumes than female controls and MtFs in the left gyri: pre- and postcentral; posterior cingulate; and calcarine as well as the precuneus regions. These observations indicate that certain regions of the left hemisphere are masculine in FtMs. But the statistical maps were uncorrected.

**Table 6:** The cortex, subcortical structures, and white matter microstructure of untreated homosexual female-to-male transsexuals

Brain region/aspect	Normative sex differences (M vs. F)	Sample size and age (*M* ± *SD* or range)	Sample source	Brain-relevant sample characteristics	Brain imaging methods and measurements	Statistically controlled confounding variables	Findings and conclusions	Authors
Cortex	F > M	10 MtF 7 FtM 7 M 11 F MtFs: 228.8 ± 7.69. FtM: 24.8 ± 6.45 M: 27.1 ± 5.54. F: 23.9 ± 3.424	Psychiatry and Psychotherapy Department, Semmelweis University, Budapest, Hungary	Ho FtMs	3 T Philips Achieva scanner VBM Cluster threshold: 30 voxels Uncorrected *Measurements* Volume	Age	M > F: Right posterior cingulated; precuneus F > M: Right superior temporal gyrus F&MtF<M& FtM: Left pre-postcentral gyrus, posterior cingulated, calcarine gyrus, and precuneus F&MtF>FtM&M: Right occipital lobe, middle inferior occipital, fusiform, and lingual gyri Right inferior temporal gyrus *Conclusions* structural differences between transsexuals and controls same biological gender	Simon et al. (2013)
Cortex	F > M	17 untreated androphilic FtM adolescents 11 untreated MtF 44 M adolescents 52 F adolescents FtMs: 15.20 ± 2.76 MtFs: 13.77 ± 2.42 M: 16.42 ± 2.75 F: 16.29 ± 2.96	Center of Expertise on Gender Dysphoria, VU University Medical Center, Amsterdam, Netherlands	Untreated androphilic adolescent FtM	3 T Philips Intera scanner VBM Clusters threshold >10 voxels FEW corrected *Measurements* Volume	Tanner stage Age IQ	FtM > F: medial frontal cortex *Conclusions* The volume of cortex in adolescent FtMs differs from F in the frontal cortex	Hoekzema et al. (2015)
Cortex Putamen	F > M	18 Hom MtFs 24 Hom FtM 29 Het M 23 Het F Hom MtF: 25.50 ± 6.91 Hom FtM: 26.21 ± 9.50 Het M. 29.28 ± 6.35 Het F: 31.09 ± 8.64	Hospital Clinic, Barcelona, Spain	Early GD onset Untreated FtMs	3 T TIM TRIO Siemens scanner analysis by FreeSurfer FEW corrected *Measurements* Cortical thickness	Age	FtM = F F > M: Left inferior parietal; Right postcentral; Right pars triangularis FtM>M: Inferior and superior parietal, superior temporal, postcentral, supramarginal Putamen: FtM = M *Conclusions* FtMs=F FtM thicker (feminine) CTh than M but differed from M in regions that F did not (Fig. 2). Their putamen is masculine	Zubiaurre-Elorza et al. (2013)
White matter	M > F	18 Hom FtMs 19 Het F 24 Het M FtM: 28.24 ± 10.61 Het F: 31.22 ± 6.09 Het M: 33.00 ± 8.22	Hospital Clinic, Barcelona, Spain	Early GD onset Untreated FtMs	3 T TIM TRIO Siemens scanner *Measurements* FA	Age	M > F: Right and left Superior longitudinal fasciculus, Forceps minor, Corticoespinal tract FtM = M: Right Posterior longitudinal , Forceps minor and corticospinal tract fasciculi *Conclusions* The main fascicles of the right hemisphere are masculine in FtMs (Fig. 3b)	Rametti et al. (2011a)

*ICV* intracraneal volume, *GM* gray matter, *WM* white matter, *CSF* cerebrospinal fluid (includes ventricles and cisterns), *FDR* false discovery rate, *VBM* voxel-based morphometry, *FEW* family-wise error correction, *CTh* cortical thickness, *FA* fractional anisotropy, *GD* gender dysphoria, *FtM* female-to-male transsexuals, *MtF* male-to-female transsexuals, *Hom* homosexual, *Het* heterosexual, *M* male, *F* female, *IQ* intelligence quotient

Untreated androphilic adolescent FtMs have also been studied (Hoekzema et al., 2015), and they show less volume in the left superior medial frontal cortex than control females and less in the right insula than control males; this study shows that certain regions of the cortex of adolescent FtMs are different from both male and female controls.

There is only one study of cortical thickness in early-onset homosexual FtMs (Table 6; Fig. 2c). Their CTh does not differ statistically from female controls but it does differ significantly from some regions in male controls in which male and female controls do not differ in CTh. Contrary to control females, FtMs showed significantly greater CTh than males in the left parieto-temporal cortex but, unlike control females, they did not differ from control males in the prefrontal orbital region (Fig. 2c; Zubiaurre-Elorza et al., 2013).

With respect to subcortical structures (Table 6), the volume of the putamen is larger in male than in female controls, but in homosexual FtMs the volume is masculinized, being similar to that of control males and differing from the volume of control females (Zubiaurre-Elorza et al., 2013).

White matter microstructure has been studied in homosexual FtMs using DTI (Table 6). It was reported that brain bundles involved in cognitive and emotional behavior were masculinized in homosexual FtMs (Fig. 3b). Males have greater FA values than female controls. FtM FA values are significantly greater than those of female controls and similar to those of male controls in the anterior and posterior right SLF and Fm. However, their CST is defeminized; that is, FtM FA values lie just between male and female controls and are significantly different from each of these two groups (Rametti et al., 2011a).

#### Conclusions

In FtMs, the gross morphological parameters correspond to their natal sex; their cortex is generally feminine but differs from males in different regions than do control females (compare Fig. 2a and c). Furthermore, some brain bundles are masculinized (Fig. 3b). All these findings suggest that homosexual FtMs have their own phenotype with respect to cortical thickness, subcortical structures, and white matter microstructure. Moreover, these changes are mostly seen in the right hemisphere.

### The Brain of Untreated Male-to-Female and Female-to-Male Transsexuals from Mixed Samples of Homosexual and Nonhomosexual Subjects

Some studies in the literature have used groups of mixed samples of MtFs in regard to their sexual orientation and this aspect was also unspecified in their control groups (Luders et al., 2009b, 2012). Others mix homosexual and nonhomosexual MtFs and FtMs and use a gathering of heterosexual, homosexual, and bisexual subjects as controls (Hahn et al., 2015; Kranz et al., 2014). These studies are very difficult to interpret and any comparison with the structural data presented in the previous sections, studying homogeneous groups of homosexual or nonhomosexual MtFs or FtMs, could confuse the picture of the brain structure of MtFs and FtMs in the context of the expression of sex differences. Nevertheless, they are summarized in Table 7.

**Table 7:** Gray and white matter in studies with mixed samples of untreated homosexual and nonhomosexual male-to-female and female-to-male transsexuals

Brain region/aspect	Normative sex differences (M vs. F)	Sample size & age (M ± SD or range)	Sample source	Brain-relevant sample characteristics	Brain imaging methods and measurements	Statistically controlled confounding variables	Findings and conclusions	Authors
Cortex	F > M	24 MtF 30 M 30 F F and M from the Consortium of Brain Mapping data base MtF: 23-72 M: 23–69 F: 23–73	MtFs: volunteers from local transsexual community, Los Angeles, USA	A mixed sample of untreated Hom and Nonhom MtFs	1.5 T Siemens Sonata scanner VBM Corrected by FDR *Measurements* Volume	Age	Cortex: F > M and MtFs MtFs = M Putamen: MtF > M *Conclusions* Gray matter in MtFs is more similar to the pattern of M than F	Luders et al. (2009b)
Cortex	F > M	24 MtFs (the same Ss as in Luders et al. (2009b) M from the Consortium of Brain Mapping data base MtFs: 45.7 ± 13.8 M: 45.9 ± 13.7	MtFs volunteers from local transsexual community, Los Angeles, USA	Mixed sample of untreated Hom and Nonhom MtFs 6 MtFs: attracted to men and 18 attracted to women No control F group	1.5 T Siemens Sonata scanner CTh by own developed strategy of analyses *Measurements* CTh	Age	MtFs>M: Left: orbitofrontal cortex;middle frontal gyrus Right: pre- and post central gyrus; temporal cortex; parietal cortex; precuneus; lingual gyrus *Conclusions* MtFs have a thicker CTh than M	Luders et al. (2012)
White matter	M > F	21 MtF (5 Hom; 4 Nonhom; 12 Bi) 22 M (16 Het; 6 Hom) 23 F (15 Het; 5 Hom; 3 Bi) 23 FtM (19 Ho; 1 Nonhom; 3 Bi) MtFs: 30.86 ± 8.38 M: 25.45 ± 4.76 F: 25.96 ± 6.07 FtM: 25.91 ± 6.83	Department of Obstetrics and Gynaecology, Medical University, Vienna, Austria	Mixed sample of Hom, Nonhom and Bi MtFs, FtMs Mixed sample of Het, Hom, and Bi M and F controls GD onset before or at puberty	3 T TIM Trio Siemens scanner DTI; TBSS *Measurements* FA MD	Age ICV	MD:F>FtM>MtF > M *Conclusions:* differences between groups in almost all white matter tracts	Kranz et al. (2014)
Connectivity		The same as in Kranz et al. (2014) + 2 M	Department of Obstetrics and Gynaecology, Medical University, Vienna, Austria	Mixed sample of Hom, Nonhom and Bi MtFs, FtMs Mixed sample of Het, Hom, and Bi M and F controls GD onset before or at puberty	3 T TIM Trio Siemens scanner DTI Probabilistic tractography *Measurements:* Several measures from graph theory	Age ICV	Increased inter-hemispheric lobar connectivity weights in MtFs and e intra-hemispheric decreases in FtMs *Conclusions* MtFs and FtMs show phenotypic characteristics in structural connectivity	Hahn et al. (2015)

*ICV* intracraneal volume, *GM* gray matter, *WM* white matter, *FDR* false discovery rate, *VBM* voxel-based morphometry, *CTh* cortical thickness, *DTI* diffusion tensor imaging, *TBSS* tract-based statistics, *MtF* male-to-female transsexuals, *FtM* female-to-male transsexuals, *M* male, *F* female, *Hom* homosexual, *Nonhom* nonhomosexual transsexuals, *Bi* bisexual, *Het* heterosexual

The study of mixed samples implicitly assumes that transsexuals are a homogeneous group. This is far from the truth with respect to the onset of GD and sexual orientation (Blanchard, 1989a, 1989b). Moreover, sexual orientation is associated to different body phenotypes. Homosexual MtFs are shorter than men in the general population, whereas nonhomosexual MtFs have been reported to be similar in height to control males (Blanchard, Dickey, & Jones, 1995). Although a later study found no significant differences regarding body mass between homosexual and nonhomosexual MtFs, other distinctive developmental and behavioral characteristics (age of onset; cross-dressing, having been married, cross-gender appearance) have been described for each subtype of transsexual (Smith et al., 2005). Finally, from the studies of Savic’s group, we know that homosexual persons show phenotypic characteristics in cortical and subcortical structures. Homosexual males and heterosexual females had thinner cortices primarily in visual areas and smaller thalamus volumes than heterosexual males (Abé, Johansson, Allzén, & Savic, 2014). Moreover, in contrast to heterosexual males, and in congruence with heterosexual females, homosexual males displayed hypothalamic activation in response to a putative male pheromone (Savic, Berglund, & Lindstrom, 2005). These observations signify that control groups in studies of the transsexual brain must be homogenous in regards to sexual orientation. Nevertheless, it is possible to extract some data from these studies if we compare those that use the same MRI techniques and measurements.

The volumetric study by Luders et al. (2009b) found that the pattern of GM variation in MtFs was more similar to the pattern found in men than in women (Table 7). They studied a mixed sample of homosexual and nonhomosexual MtFs. The Savic and Arver (2011) volumetric study of nonhomosexual MtFs reported that their brains were not feminized. It could be that in Luders et al.’s (2009b) study there were more nonhomosexual than homosexual MtFs and, as a result, the two studies reached the same conclusion, in contrast to the volumetric study of Simon et al. (2013) that reported cortical feminization in homosexual MtFs.

It is practically impossible to compare the two studies on CTh in MtFs because of the differences in their samples and designs. Luders et al. (2012) did not specify sexual orientation in either their transsexual or their control group and used only males as controls, while Zubiaurre-Elorza et al.’s (2013) study design employed male and female heterosexual controls to study homosexual MtFs.

In regard to white matter microstructure, the study of Kranz et al. (2014) mixed sexual orientation within their MtF, FtM, and female and male control groups (Table 7). The differences in design and sampling make it almost impossible to compare these studies with those of our group (Rametti et al., 2011a, 2011b; see Tables 5, 6, 7). However, since in Kranz et al.’s study most of the FtMs were homosexuals (19/24), it could feasibly be compared with the results of Rametti et al. (2011a) presented in the previous section (Table 6). Kranz et al. did not find FA differences between FtMs and control groups but they did find significantly decreased MD values in FtMs with respect to control females in the same tracts with increased FA values in FtMs (Rametti et al. 2011a; Table 6). Thus, MD results also indicate a defeminization or masculinization of the white matter microstructure in FtMs, as was reported by Rametti et al. (2011a).

With the same design and sample as in Kranz et al., structural connectivity has been studied from DTI using graph theory (Hahn et al., 2015). The study reported that FtMs, with respect to male and female controls and MtFs, have decreased intra-hemispheric connectivity between the right subcortical/limbic and right temporal lobes (Table 7). Interestingly, the changes in brain connectivity found in FtMs and MtFs are in opposite directions and are only seen in the right hemisphere.

Recently, resting-state fMRI was used to study the similarities between spontaneous brain connectivity in one untreated FtM of unspecified GD onset and sexual orientation with polycystic ovary syndrome and male and female controls. This FtM subject showed a functional connectivity profile that was comparable to that of the subject’s natal sex (Santarnecchi, Vatti, Dettore, & Rossi, 2012).

### Theoretical and Functional Implications of the Brain Phenotype of Untreated Homosexual Transsexuals

Untreated homosexual MtFs and FtMs show a complex picture for the expression of sex differences in their brains (Tables 5, 6). Contrary to some popular ideas, the MtF brain is not completely feminized but presents a mixture of masculine, feminine, and demasculinized traits. This is better illustrated by the data on CTh and FA (Table 8). Moreover, the brain of homosexual FtMs is not uniformly masculinized but presents a mixture of feminine, defeminized, and masculinized morphological traits (Table 9). For both MtFs and FtMs, the morphological traits observed depend on the region and the type of measurement taken. Thus, the morphology of the brain of homosexual MtFs and FtMs strongly suggests that each one has its own phenotype, and that the phenotype is different from those of heterosexual males and females.

**Table 8:** The brain phenotype of untreated homosexual male-to-female transsexuals from studies of cortical thickness and white matter microstructure

	Normative sex differences (M vs. F)	Phenotype	Hemisphere
Cerebral compartments	M > F	Masculine	
Gray matter	M > F	Masculine	
White matter	M > F	Masculine	
Intracranial volume	M > F	Masculine	
CSF			
Cortical thickness	F > M	Feminine	Right
Global	F > M	Feminine	Right
Orbitofrontal	F > M	Feminine	Right
Insular	F > M	Feminine	Right
Cuneus			
White matter microstructure			
Longitudinal superior	M > F	Demasculinized	Right
Fronto-occipital inferior	M > F	Masculine	
Forceps minor	M > F	Demasculinized	Right
Cingulum	M > F	Demasculinized	Right
Corticospinal tract	M > F	Demasculinized	Right

This table summarizes findings shown in Table 5. See Rametti et al. (2011b) and Zubiaurre-Elorza et al. (2013)

**Table 9:** The brain phenotype of untreated homosexual female-to-male transsexuals from studies of cortical thickness and white matter microstructure

	Normative sex differences (M vs. F)	Phenotype	Hemisphere
Cerebral compartments			
Gray matter	M > F	Feminine	
White matter	M > F	Feminine	
Intracranial volume	M > F	Feminine	
CSF	M > F	Feminine	
Cortical thickness			
Global	F > M	Feminine	Right
Parieto-temporal	F > M	Feminine	Right and left
Parietal	F > M	Feminine	Right
Subcortical structures			
Putamen (volume)	M > F	Masculine	Right
White matter microstructure			
Longitudinal superior	M > F	Masculine	Right and left
Forceps minor	M > F	Masculine	Right
Corticospinal tract	M > F	Defeminized	Right

This table summarizes findings shown in Table 6. See Rametti et al. (2011a) and Zubiaurre-Elorza et al. (2013)

In terms of psychological presentation, people with an early onset of GD have much in common with individuals with somatic intersexuality (Meyer-Bahlburg, 2011). None of the above neuroimaging studies included subjects with signs of somatic intersexuality. MtFs and FtMs each have their own cerebral phenotype. This would suggest that early-onset homosexual transsexuals have an intersex condition restricted to the brain. “Brain hermaphroditism” was suggested in the early postmortem studies (Kruijver, Zhou, Pool, Hofman, Gooren, & Swaab, 2000).

It has been pointed out that verifying this hypothesis requires corroborating at least one of the genetic, hormonal, or morphological lines of research into some specific effect by hormones that would affect brain organization of sexual differences but not other organs (Meyer-Bahlburg, 2011, 2013). The existing findings on brain changes fulfill at least one of the conditions and provide evidence that the brain structures are already affected in still untreated homosexual MtFs and FtMs. Moreover, in androgenized FtMs, FA value increases in the SLF and the CST can be predicted by the free testosterone index before the treatment begins (Rametti et al., 2012). All these findings support the intersex hypothesis of transsexuality.

The right-side asymmetry in the differences between MtFs, FtMs, and control males (Tables 8, 9) focuses attention on that hemisphere. Sex differences in functional hemispheric lateralization are well known. Transsexuals have been studied from this perspective, especially in relation to mental rotation and handedness, because these may be influenced by prenatal androgen levels, which would reflect some developmental anomaly. Laboratory animal experiments with rats suggest that cerebral cortical laterality differs between the sexes and that gonadectomy at birth will alter the usual cortical laterality (Diamond, 1991).

The right hemisphere is involved in mental rotation and males outperform females (Voyer, Voyer, & Bryden, 1995). Mental rotation performance in untreated early-onset homosexual MtFs and FtMs is consistent with that of their natal sex and not with that of their gender identity (Haraldsen, Opjordsmoen, Egeland, & Finset, 2003). This is also reflected by the finding that untreated MtFs perform better than untreated FtMs in these tasks (Slabbekoorn, van Goozen, Gooren, & Cohen-Kettenis, 2001). However, there are reports in which transsexual groups show a pattern of performance that is different from their biological sex (Cohen-Kettenis, van Goozen, Doorn, & Gooren, 1998). The pattern of brain activation in mental rotation involves the frontal, parietal, and posterior occipital regions (Carrillo et al., 2010; Cohen et al., 1996; Hugdahl, Thomsen, & Ersland, 2006). Untreated MtFs and FtMs show parietal activation (Sommer et al., 2008). The fronto-parietal-occipital pattern of activation is also seen in early-onset homosexual MtFs and FtMs after long-term cross-sex hormone treatment (Carrillo et al., 2010). At present, it is not possible to relate changes observed in the right cortex and fascicles of transsexuals (Tables 8, 9) with differences in mental rotation abilities. However, the superior longitudinal fasciculus, which connects fronto-parietal regions, is demasculinized in MtFs and masculinized in FtMs. Moreover, the right parietal region is thicker in FtMs than in males, while MtFs present a thicker cortex than males in visuoperceptive occipital regions (cuneus and pericalcarine regions) (see Fig. 2b, c). We are still far from being able to relate these morphological differences to spatial abilities in transsexuals.

Hand preference has also been studied in transsexuals (Green & Young, 2001). Sex differences in hand preference are well known, left-handedness being more common in males than females (McGlone, 1980). In young boys and girls, prenatal testosterone exposure was related to a decrease in strength of handedness (Lust et al., 2011). Pre-pubertal boys with GD were more often left-handed than control males (Zucker, Beaulieu, Bradley, Grimshaw, & Wilcox, 2001). This is also seen in adult MtFs and FtMs before (Cohen-Kettenis et al., 1998) and after cross-sex hormone treatment (Green & Young, 2001; Orlebeke, Boomsma, Gooren, Verschoor, & Van Den Bree, 1992; Watson & Coren, 1992; Wisniewski, Prendeville, & Dobs, 2005). This would suggest a different pattern of cerebral hemispheric organization in transsexuals.

The CST is the most important motor tract with fibers originating in motor, premotor, and sensory cortices (Lemon, 2008). Studies using DTI techniques have shown that the CST exhibits a leftward asymmetry (Dubois et al., 2009; Westerhausen, Huster, Kreuder, Wittling, & Schweiger, 2007) that is present as early as 4 months of life (Dubois et al., 2009) and seems to be unrelated to hand preference (Nathan, Smith, & Deacon, 1990; Westerhausen et al., 2007). However, a study of the CST in 400 adolescents (12–18 years old) found that this tract, at the level of the internal capsule, shows a strong left > right hemispheric asymmetry that is less marked in left-handed subjects (Herve et al., 2009). Moreover, CST increases in this region in males but not in females, so the increases must be related to the plasma levels of testosterone (Herve et al., 2009). Using DTI techniques, it was found that early-onset homosexual MtFs have a demasculinized CST (Rametti et al., 2011b), while, in early-onset homosexual FtMs, this tract is masculinized (Rametti et al., 2011a). DTI studies of CST microstructure in transsexuals are a first step and signal a new direction for future explorations of hand preference in transsexuals.

Results on cortical thickness also suggest that this parameter would be a good target for a systematic study on body perception mechanisms in MtFs and FtMs. The right hemisphere is mainly involved in the analysis of body perception and its emotional connotations (Longo, Azanon, & Haggard, 2010). It was underscored above that the main brain differences shown by transsexuals are located in the right hemisphere. Generally, the emergence of a masculine or feminine identity must be strongly mediated by the early development of a male or female body self-perception. This requires several levels of construction of somatoperception and somatorepresentation; the latter includes emotions, attitudes directed towards one’s own body, and the link between the physical body and the psychological self (Longo et al., 2010). The body model of identity integrity would implicate a right fronto-parietal and insular network (Giummarra, Bradshaw, Nicholls, Hilti, & Brugger, 2011) and differences have been reported for homosexual MtFs and FtMs in all these regions (Zubiaurre-Elorza et al., 2013).

The literature on body perception in transsexuals reflects two approaches. One comes from an analysis of the desire to amputate a limb as a type of identity disorder (First, 2005) and the other is a theoretical hypothesis generated from the analysis of the phantom limb phenomenon (Ramachandran & McGeoch, 2007).

It has been suggested that the desire for limb amputation could be similar to transsexualism because in most cases the goal of amputation is to match one’s body to one’s identity (First, 2005). The similarities with transsexuals are mainly associated with the feeling of being uncomfortable with an aspect of one’s anatomical identity. It should be remembered that some transsexuals not only reject the masculine or feminine aspects of their bodies but they dislike specific regions (i.e., breasts in FtMs and genitals in MtFs). This body uneasiness experienced by transsexuals diminishes after cross-sex hormone treatment (Fisher et al., 2014). In addition, the desire for limb amputation has an early onset in childhood or adolescence and a significant subgroup of these individuals experiences sexual arousal by fantasizing about the desired limb amputee identity (First, 2005).

The theoretical parallel between the desire for limb amputation and transsexuality has been analyzed by Lawrence (2006). Nonhomosexual but not homosexual MtFs seem to share some characteristics with those who desire limb amputation. It should be noted that in most cases subjects want a left-limb amputation (First, 2005) and this may reflect some dysfunction in the right hemisphere, precisely the hemisphere in which homosexual MtFs and FtMs present differences with controls (Zubiaurre-Elorza et al., 2013).

Based on amputation studies and provisional data on the phantom limb phenomenon after penis or breast amputation in transsexuals, it was hypothesized that during embryological development the brain of transsexuals was hard-wired in a manner that was opposite to that of their natal sex (Ramachandran & McGeoch, 2007). No posterior study has verified this hypothesis nor have the preliminary data been published or shown by the authors.

Recently, a new strategy has been employed (Feusner et al., 2016). Homosexual/bisexual FtMs viewed photographs of their own body that were morphed by different degrees to bodies of other females or males and were instructed to rate “To what degree is this picture you?” FtMs differed from heterosexual male and female controls because they rated body images as more self-like when they were morphed to the sex congruent with their gender identity rather than to their natal sex.

Cerebral circuitry involved in body perception has been studied. The inferior parietal and premotor cortices play a role in perceptual judgment about body configuration; the insular lobes are involved in body awareness in general and the right insula in egocentric representation, self-recognition, and body ownership (Tsakiris, 2010). Circuitry and connectivity analyses have revealed the afferent and efferent connectivity of the insula, the somatosensorial, the temporo-parietal, and the premotor cortices. MRI and neuropsychological data favor a right hemispheric specificity for self-processing in general and for body ownership specifically. We have seen that MtFs differ from males in visuoperceptive regions such as the cuneus and calcarine region as well as in regions related with body perception and emotional experience of the body (insula) and reward value (medial orbitofrontal cortex; Fig. 2b). Moreover, some fascicles related to these regions are demasculinized (Fig. 3a). On the other hand, FtMs differ from males in the parietal and postcentral regions of the right hemisphere and have masculine fascicles related to these regions in the right frontal lobe (Figs. 2c, 3b). Thus, the available structural data show specific differences for MtFs and FtMs in cortical regions and fascicles involved in body perception.

Savic and Arver (2011) found that nonhomosexual MtFs have larger gray matter volume than male and female controls in the right parieto-temporal junction and the right inferior frontal and the insular cortices. As shown above, these regions are related to body self perception. The authors suggested that the experience of dissociation of the self from the body may be a result of failure to integrate complex somatosensory and memory processes in these regions. Future research should explore possible differences in the structural connectivity of these regions.

Differences have been detected in the neural network of body representation in transsexuals (Lin et al., 2014). Lin et al. investigated the regional changes in the degree of centrality in resting-state functional connectivity of the brain; the degree of centrality is an index of the functional importance of a node in a neural network. They hypothesized that three key regions of the body representation network (primary somatosensory cortex, parietal lobe, and insula) would show a higher degree of centrality in untreated transsexuals with respect to controls. Transsexuals do show a higher degree of centrality in the bilateral parietal lobe and the somatosensory cortex. However, their data analysis pooled the data from MtFs and FtMs. Although the findings of Lin et al. are indicative of specific connectivity features in transsexuals, they should be taken cautiously until separate analyses distinguishing between MtFs and FtMs, the onset of the GD, and sexual orientation can be presented.

### Comment on the Brain of Nonhomosexual Transsexuals

As noted above, there is only one morphological study on untreated nonhomosexual transsexuals in the literature (Savic & Arver, 2011). This study and our proposed phenotypes for homosexual MtFs and FtMs could help us take the first steps in discerning between homosexual and nonhomosexual transsexuals. Homosexual MtFs are female-like in a series of sexually dimorphic behaviors, while nonhomosexual MtFs are not (Blanchard, 1989a, 1989b). It has also been hypothesized that the brain of homosexual and nonhomosexual MtFs would differ from that of males in different ways. In homosexual MtFs, the differences would involve sexually dimorphic structures and the nature of the differences would be a shift toward the female-typical patterns, while in nonhomosexual MtFs the differences themselves would not involve sexually dimorphic structures (Blanchard, 2008). Moreover, it was also suggested that “if there is any neuroanatomic intersexuality, it is in the homosexual group” (Blanchard, 2008).

Following this line of thought, Cantor (2011, 2012, but also see Italiano, 2012) has recently suggested that Blanchard’s predictions have been fulfilled in two independent structural neuroimaging studies. Specifically, Savic and Arver (2011) using VBM on the cortex of untreated nonhomosexual MtFs and another study using DTI in homosexual MtFs (Rametti et al., 2011b) illustrate the predictions. Cantor seems to be right. Nonhomosexual MtFs present differences with heterosexual males in structures that are not sexually dimorphic (Savic & Arver, 2011), while homosexual MtFs (as well as homosexual FtMs) show differences with respect to male and female controls in a series of brain fascicles (Rametti et al., 2011a, 2011b). If other VBM and CTh studies on the cortex of homosexual MtFs are added (Simon et al., 2013; Zubiaurre-Elorza et al., 2013), there is a more substantial number of untreated homosexual MtFs and FtMs that fulfill Blanchard’s prediction but still only one study on nonhomosexual MtFs; to fully confirm the hypothesis, more independent studies on nonhomosexual MtFs are needed. A much better verification of the hypothesis could be supplied by a specifically designed study including homosexual and nonhomosexual MtFs.

Finally, for Blanchard, MtF and FtM homosexual transsexuality is an extreme expression of homosexuality. He considered the following continuum: homosexual → gender dysphoric homosexual → transsexual homosexual (Blanchard, Clemmensen, & Steiner, 1987). Later, Blanchard also hypothesized that homosexual transsexuals should show differences in sexually dimorphic brain structures (Blanchard, 2008). Thus, from Blanchard’s view, there would be no brain differences between homosexual transsexuals and homosexual persons. This hypothesis has not been directly tested yet. However, there are two studies in the literature with respect to cortical thickness that, taken cautiously, may approach Blanchard’s hypothesis on the relationship between transsexuality and homosexuality.

The only study on the CTh of homosexual persons that do not present gender dysphoria is by the Savic group (Abé et al., 2014). If we compare this study with that of Zubiaurre-Elorza et al. (2013) on the CTh of homosexual MtFs, we see both studies report sex differences showing an F > M pattern in similar structures of the right hemisphere. But there is only one region, the *pars triangularis*, in which homosexuals and homosexual MtFs both present differences. However, these changes are in opposite directions. The pars triangularis of homosexual MtFs is thicker than in heterosexual male controls, while for homosexuals it is thinner than in heterosexual males. Thus, it seems that for transsexuals this region is feminized but demasculinized in homosexual individuals. Interestingly, in both studies, the affected pars *triangularis* is in the right hemisphere. Nevertheless, confirming Blanchard’s prediction still needs a specifically designed comparison of homosexual MtF, homosexual male, and heterosexual male and female people.

## The Transsexual Brain Phenotype in the Light of the Neurohormonal Theory of Brain Sexual Differentiation

The cortex of homosexual MtFs and FtMs is feminine and has an F > M morphological pattern of sex differences. Nevertheless, the FA of brain fascicles is either demasculinized (MtFs) or masculinized/defeminized (FtMs), while in control groups, sex differences in the FA show an M > F pattern (see Tables 8, 9). Fortunately, animal models have provided information on the hormonal mechanisms implicated in the development of the M > F and F > M patterns. This information helps explain the role of hormones in creating the different human sexual brain phenotypes.

The F > M pattern in the cortex of MtFs, FtMs, and females is also present in structures of the rat brain such as the medial anterior region of the bed nucleus of the stria terminalis (BSTMA), lateral anterior region of the bed nucleus of the stria terminalis (BSTLA), anteroventral periventricular nucleus (AVPv), arcuate nucleus (Arc), parastrial nucleus (PS), and the locus coeruleus (LC) (Guillamon & Segovia, 1996). Neonatal orchidectomy in males increases the morphological measurements (volume and/or number of cells) of the BSTMA (del Abril, Segovia, & Guillamon, 1987), BSTLA (Guillamon, Segovia & del Abril, 1988), and AVPv (Davis, Shryne, & Gorski, 1996) to levels similar to those of females. However, early postnatal androgenization of females decreased the morphological measurements in the BSTMA and BSTLA (del Abril et al., 1987; Guillamon, Segovia & del Abril, 1988). In consequence, it was suggested that the smaller measurements of males were due to an “inhibitory” action by androgens (Segovia & Guillamon, 1993).

This inhibitory effect is supported by a study comparing the volume and the number of neurons in the LC of rats with testicular feminization syndrome (Tfm) to that in their male littermates. In the LC, Tfm rats lack functional AR and have a larger volume and greater number of neurons than their control male littermates (Garcia-Falgueras et al., 2005). However, the inhibitory mechanism may vary depending on the region, since ER seems to mediate neuronal cell death in the AVPv (Waters & Simerly, 2009). Curiously, the development of sex differences in the AVPv and LC nuclei occurs postpuberally (Davis et al., 1996; Pinos et al., 2001), indicating the importance of puberty in the development of the F > M pattern.

This inhibitory androgen action also operates in the human cortex. It has been shown that the possession of an allele conferring more efficient function on the AR is associated with a thinner cortex (Raznahan et al., 2010) and a relatively thinner and less dense gray matter (Paus et al., 2010) in adolescents. The thicker cortex observed in specific regions of the cortices of MtFs and FtMs could be explained by an atypical regional functioning of the testosterone-receptor complex that would be constrained to particular regions of the brains of MtFs and FtMs (compare Fig. 2b and c). A possible explanation could be differential gene expression produced by some epigenetic process that would affect the AR and/or their equilibrium with ER in certain regions of the cortex of transsexuals.

Human white matter shows sex differences that follow the M > F pattern. Males have a greater increase in brain white matter during childhood and adolescence than females (De Bellis et al., 2001; Lenroot et al., 2007; Perrin et al., 2008). The human white matter microstructure, as measured by FA, is also M > F. This pattern has been the most reported in animal studies. Testosterone is responsible for promoting the greater volume and number of neurons seen in males, as has been shown in the nuclei constituting the neural network of the accessory olfactory system in the rat (Guillamon & Segovia, 1996). Neonatal male gonadectomy decreases the volume and number of neurons in these nuclei, while neonatal androgenization of the female increases the volume and number of neurons.

The role of testosterone and the AR has been studied in relation to the growth of the white matter in adolescents. The testosterone-related increase in white matter volume was stronger in adolescents with fewer CAG repeats in the androgen receptor gene (Perrin et al., 2008); fewer CAG repeats make androgen receptors more effective. The masculinization of FA observed in some brain fascicles of FtMs before cross-sex hormone treatment seems to be related to testosterone (Table 9; Fig. 3b). This is because the increments in FA values in the SLF and CST tracts are predicted by the free testosterone index before hormonal treatment (Rametti et al., 2012). Brain fascicles are demasculinized in MtFs (Table 8; Fig. 3a) and this might be related to atypical function of testosterone or the androgen receptor.

Examining the two morphological patterns of sexual dimorphism together, general rules emerge. In structures with the F > M pattern, testosterone inhibits development in both males and females. The fact that MtFs and FtMs show atypical development in specific cortical regions suggests that this rule does not hold in the particular cortical regions in which homosexual MtFs and FtMs differ from male and female controls. In structures with the M > F pattern, testosterone promotes growth but this rule again does not hold for the white matter microstructure of some brain bundles in MtFs and FtMs.

## A Unifying Neurodevelopmental Hypothesis to Explain the Expression of Sex Differences in Untreated Homosexual Male-to-Female and Female-to-Male Transsexuals

The phenotype for cortical thickness has a common feature in homosexual MtFs, FtMs, and females; in all three cases, the morphological pattern is F > M. This observation could help provide a hypothesis that supplies a single explanation for the cortical phenotype in MtFs, FtMs, and male and female controls. We acknowledge that our hypothesis is still tentative because of the paucity of studies on CTh in homosexual transsexuals and of the limitations inherent to MRI techniques.

The hypothesis emerges from a developmental approach to the cortex. Why the cortex? First, focusing attention on the cortex is quite justified in studying gender identity because of its integrative and commanding functions with respect to all types of behaviors and its interconnectivity with subcortical structures. Second, the most marked and most consistent differences found in transsexuals’ brains are seen in the cortex (Zubiaurre-Elorza et al., 2013). Third, the cortex contains receptors for androgens (Beyenburg et al., 2000; Finley & Kritzer, 1999; Puy et al., 1995), and α- and β-estrogen (Gonzalez et al., 2007; Montague et al., 2008; Osterlund, Gustafsson, Keller, & Hurd, 2000). Fourth and last, cortical sex differences follow the F > M pattern and the hormone mechanisms that control this pattern in mammals agree with the MRI developmental studies of the human cortex as shown above.

Although control females, MtFs, and FtMs all have an F > M pattern, they are different among themselves because they differ from control males in different cortical regions. As a result, each of these categories has a distinct phenotype.

The thickness of the human cortex presents an F > M pattern of sex differences (Im et al., 2006; Luders, Narr, Zaidel, Thompson, & Toga, 2006; Raznahan et al., 2010; Shaw et al., 2008; Sowell et al., 2007; Zubiaurre-Elorza et al., 2013) and it becomes thinner over the first three decades of life (Shaw et al., 2008). However, this process is not homogeneous, since cortical regions with a simple laminar architecture (limbic areas) also show simple growth trajectories (linear and quadratic), while polysensory and high-order association areas of the cortex have more complex developmental trajectories (cubic) (Shaw et al., 2008).

**Fig. 4:**
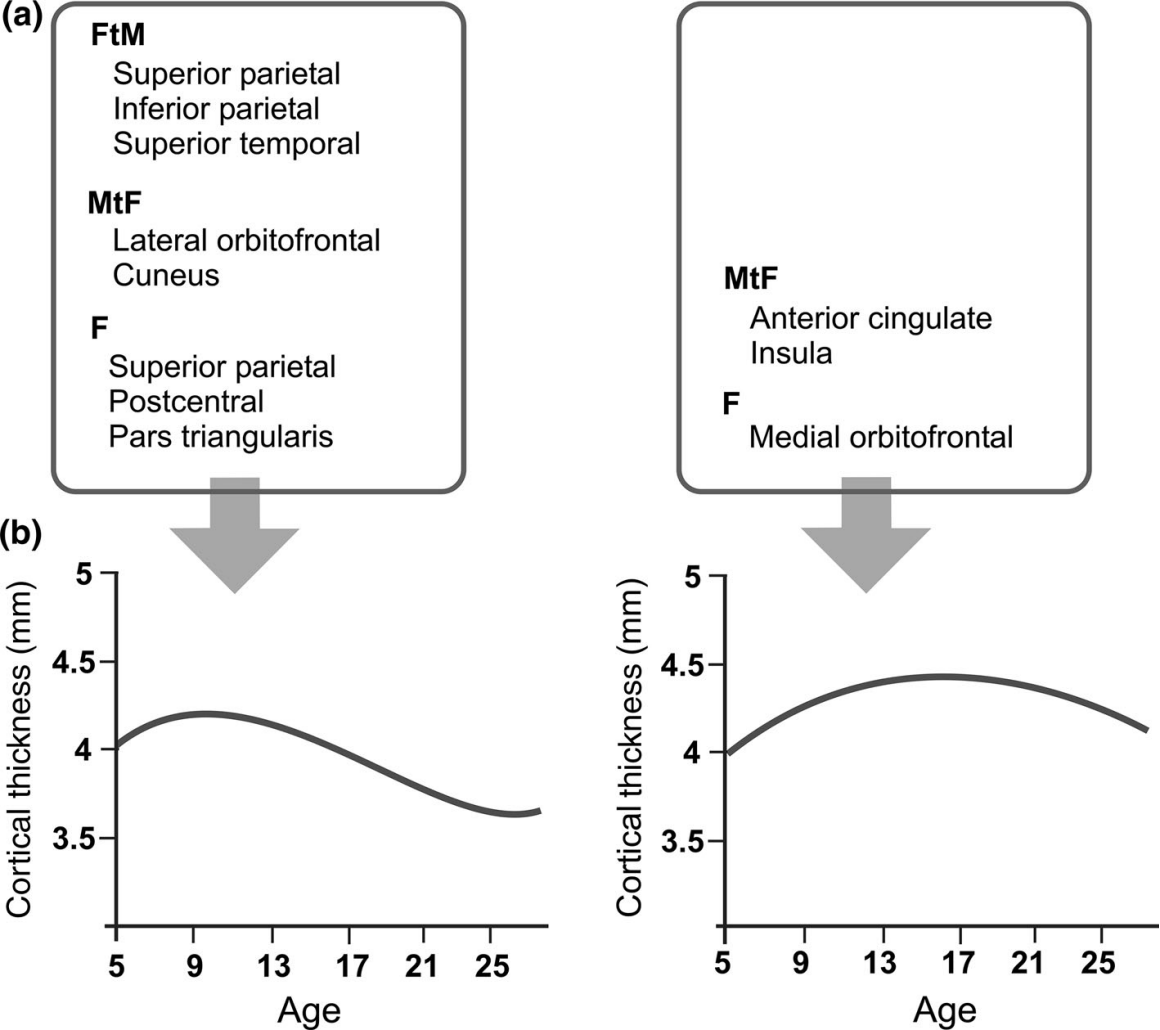
Cortical thickness developmental trajectories and changes with respect to gender. **a** Zubiarre-Elorza et al.’s (2013) findings on the regions in which cortical thicknesses in untreated male-to-female (MtF) and female-to-male (FtM) transsexuals and control females (F) are thicker than those in control males are summarized on the *top panels* over the graphs representing the developmental process for cortical thickness (**b**), described by Shaw et al. (2008). The structures in the *top left panel* are homotypical-isocortical and follow a cubic trajectory, while those in the top right panel are transitional and follow a quadratic trajectory. Note that maximum cortical thickness occurs around puberty (**b**, left) and adolescence (**b**, right)

If the figures presented by Shaw et al. (2008) on the developmental trajectories of cortical structures are compared with the findings of Zubiaurre-Elorza et al. (2013), it can be seen that females, MtFs, and FtMs differ from males (Fig. 4). In addition, the cubic and quadratic developmental trajectories show a peak in cortical thickness during puberty and adolescence.

Moreover, a recent study has shown that possession of an allele conferring more efficient functioning on the AR is associated with masculinization (thinning) of adolescent cortical thickness (Raznahan et al., 2010). Thus, sex differences in the development of cortical thickness might be mediated by AR. What seems to be clear from all these observations is that females, MtFs, and FtMs share the same developmental process that produces a thicker cortex than in males.

It should be remembered from the above animal studies that some brain nuclei present an F > M pattern of sex differences as in the human cortex, and androgens and sex hormone receptors (AR or ER) are implicated in the development of this pattern. Although there are conflicting results (Ujike et al., 2009), a significant association has been reported between longer AR gene polymorphisms and MtFs (Hare et al., 2009) and ERβ polymorphism in FtMs (Fernandez et al., 2014b; Henningsson et al., 2005).

Taking all these data together with the observation that females, MtFs, and FtMs all have an F > M pattern of sex differences with respect to their cortical thickness, we propose a slowing (or a stop) in the cortical thinning process in females, MtFs, and FtMs compared to the thinning process in males. However, this slowing of the thinning process affects different cortical regions in females, MtFs, and FtMs, and this particularity gives each of them their characteristic cortical phenotype.

The different thinning processes in different cortical regions support a unifying developmental hypothesis that explains cortical development for both MtFs and FtMs, and how it affects different areas at different times. This hypothetical process, based on differential developmental processes in specific cortical regions, would influence the development of gender identity for all: male, female, MtF, and FtM.

This hypothesis for the expression of sex differences in gender identity assumes a perinatal action by androgens (or their metabolites) but it should be remembered that the process of cortical thinning begins around puberty (Fig. 4). Puberty is an important period in gender identity (Steensma, Kreukels, de Vries, & Cohen-Kettenis, 2013), one in which adolescents may persist, desist (de Vries & Cohen-Kettenis, 2012; Drummond, Bradley, Peterson-Badali, & Zucker, 2008; Steensma, Biemond, de Boer, & Cohen-Kettenis, 2011; Wallien & Cohen-Kettenis, 2008), initiate (Zucker et al., 2012), or even desist and then return to their gender dysphoria (Steensma & Cohen-Kettenis, 2015). It should also be noted that animal models indicate that puberty is a period of brain remodeling (Schulz et al., 2009; Sisk & Zehr, 2005).

Returning to the neurohormonal theory of brain and behavior differentiation, it seems that in children who have no problems with their gender identity and those who experience GD at these ages and continue to do so after puberty (persisters), gender identity does not seem to have been affected by sex hormone activation. The existence of behavioral traits that only need the organizational action of sex hormones was mentioned above (Goy & McEwen, 1980) and is not unknown in transsexuals, for instance, the organizing but not activating effects of hormones were demonstrated in spatial tests in homosexual MtFs and FtMs (van Goozen, Slabbekoorn, Gooren, Sanders, & Cohen-Kettenis, 2002).

However, for those children whose GD fades (desisters) the activational effects of sex hormone and environmental influences during puberty might play a significant role. In these cases, hormones at puberty might act in two ways. One would be directly on the brain, affecting cortical development, and the other would be to guide the development of the secondary sex characteristics that would in turn be perceived as congruent because of the brain changes that take place at this age. Whether persisters-after-interruption could be fit into one of the two previous developmental pathways depends on whether this probably new developmental category is substantiated by future research. Moreover, one way to verify our neurodevelopmental hypothesis on gender would be to compare the CTh of young untreated adult persisters, desisters, and male and female controls of the same age.

## The Brain of Transsexuals After Cross-Sex Hormonal Treatment

### Effects of Cross-Sex Hormone Treatment on the Brain of MtFs and FtMs

The objective of cross-sex hormonal treatment is to feminize the bodies of MtFs, administering estradiol plus antiandrogens, or to masculinize the body of FtMs, administering testosterone. These treatments do not affect their gender feelings, which must be very well established to make them eligible for cross-sex hormone treatment. The treatments are intended to overcome dysphoria and increase their quality of life (Gomez-Gil, Zubiaurre-Elorza, de Antonio, Guillamon, & Salamero, 2014; Gomez-Gil et al., 2012).

Reviewing the effects of cross-sex hormonal treatment on the brain is important. Most of the studies on the effects of such treatments focus on the vascular and skin systems as well as the metabolism (Gooren, Giltay & Bunck, 2008).

A couple of studies examined the effects of cross-sex hormone treatment on the shape and area of the corpus callosum. Emory et al. (1991) using in vivo MRI techniques and a cross-sectional design in MtFs and FtMs did not find differences with respect to male and female controls. However, using a different technique, Yokota et al. (2005) reported that at the midsagittal plane callosal shapes were congruent with gender identity in presumably treated MtF and FtM individuals.

Only three published studies have addressed the effect of cross-sex hormone treatments on the brain of transsexuals in vivo using a longitudinal design; that is, studying the same people before and after treatment (Hulshoff Pol et al., 2006; Rametti et al., 2012; Zubiaurre-Elorza, Junque, Gomez-Gil, & Guillamon, 2014). These studies give a consistent picture on the effect of cross-sex hormone treatment on the brain.

With respect to MtFs, Hulshoff Pol et al. (2006) found that before treatment subjects’ brain volume was consistent with their natal sex. Estradiol plus antiandrogen produced a decrease in brain volume “towards female proportions” after 4 months of treatment, a decrease they found to be ten times the average annual decrease in healthy adults. Moreover, the ventricles became larger.

**Fig. 5:**
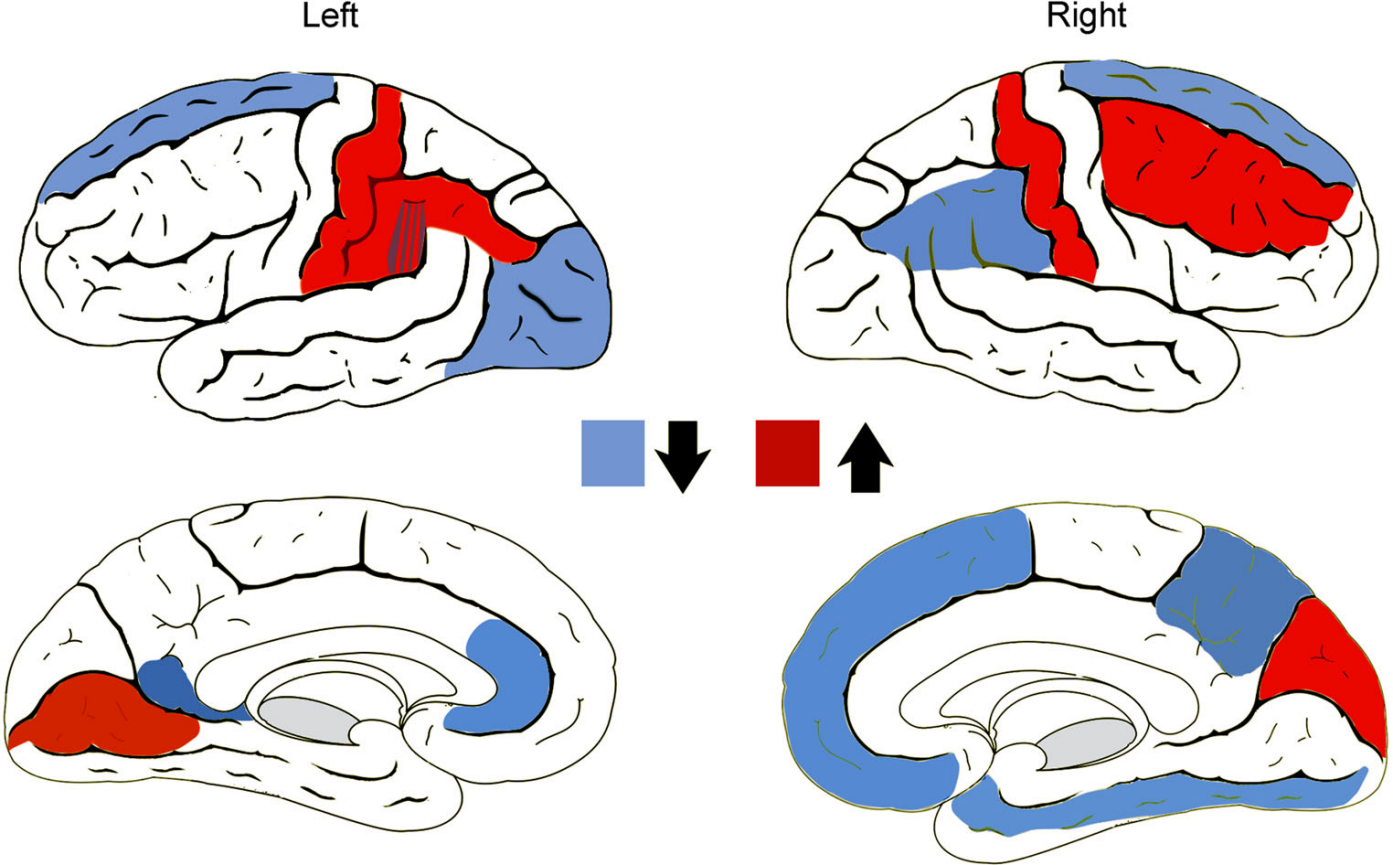
Effects of the cross-sex hormonal treatment on the cortical thickness of male-to-female (MtFs) and female-to-male (FtMs) transsexuals. *Blue*↓: cortical regions in which estradiol + antiandrogens thinned the cortex in MtFs. *Red*↑: cortical regions in which testosterone thickened the cortex in FtMs. Data transformed in images from the longitudinal study of Zubiaurre-Elorza et al. (2014)

Recently, Zubiaurre-Elorza et al. (2014) measured cortical thickness and the volume of the brain and cortical and subcortical structures in MtFs. After at least 6 months of treatment, they found a general decrease in all these measurements together with increased ventricle volume. The volumetric analyses showed a significant decrease in the total and cortical gray matter volumes. Subcortical gray matter volume also showed a decrease that was centered in the right thalamus and right pallidum. Consequently, and due to the production of a mechanical vacuum, there was a significant increase in the whole ventricular system. The decrease seen in CTh was also generalized but most strongly affected the cortex in the occipital, temporal, and parietal regions and some areas of the frontal lobes (Fig. 5).

In relation to FtMs after testosterone treatment, Hulshoff Pol et al. (2006) reported that 4 months of androgen treatment increased total brain and hypothalamus volumes. Moreover, Zubiarre-Elorza et al. (2014) reported that total (cortical + subcortical) gray matter volume increased after at least 6 months of treatment. With respect to subcortical structures, the right thalamus volume was increased. In addition, CTh increases in parietal and occipital regions of the left hemisphere were correlated with the rise in the serum testosterone and free testosterone index (Fig. 5). The androgenization treatment did not significantly affect the ventricular system of FtMs.

The effects of testosterone treatment on FA values were also studied in FtMs (Rametti et al., 2012). After seven months of treatment, FA values increased in the right SLF and the right CST fascicles compared to pre-treatment values. These increments in FA were predicted by the free testosterone index before the testosterone treatment. The higher the testosterone index before hormonal treatment, the higher the increases in FA values in the SLF and the CST fascicles under androgenization.

The above three studies suggest that cross-sex hormone treatment affects the gross morphology as well as the white matter microstructure of the brain. Changes are to be expected when hormones reach the brain in pharmacological doses. Consequently, one cannot take hormone-treated transsexual brain patterns as evidence of the transsexual brain phenotype because the treatment alters brain morphology and obscures the pre-treatment brain pattern. Thus, at least two main questions emerge from the above results. First, what mechanisms produce these alterations in the brain of MtFs and FtMs? Second, do these changes have clinical consequences?

### Possible Mechanisms That Control Brain Alterations After Cross-Sex Hormone Treatment

With respect to the mechanisms, it should be remembered that the human brain contains receptors for androgen and estrogen (see above), as well as glucocorticoids (Kicman, 2008) and, what is more, it also possesses most of the enzymes involved in the biosynthesis and metabolism of sex steroids and glucocorticoids (Österlund, Keller, & Hurd, 2000).

In relation to FtMs, it was suggested that the reported morphological increments might be due to the anabolic effects of testosterone (Zubiaurre-Elorza et al., 2014). Testosterone has virilizing and anabolic effects that are inseparable. FtMs under hormonal treatment are natal females with supraphysiological levels of testosterone that might affect the normal metabolic mechanisms of androgens. There are several nonexclusive mechanisms involving the AR and ER that could account for the morphological increments observed in FtMs. Testosterone, and its reduced metabolite dihydrotestosterone, can act directly via the AR. Moreover, some reduced metabolites from dihydrotestosterone (3α diol and 3β diol) can also bind ER. In addition, testosterone is converted to estradiol in the brain via the p450 enzyme aromatase and then binds to ER. Human brain is known to express aromatase and reductase activities (Lephart, 1996; Österlund et al., 2000; Pelletier, 2010).

There is yet another mechanism that could contribute to morphological increases in FtM brains. Androgens interfere with glucocorticoid receptor expression (Negri-Cesi, Poletti, & Celotti, 1996) producing an anticatabolic effect and a positive nitrogen balance. The view that morphological increments in the brain are due to anabolic effects is also supported by the increased FA values observed in several brain fascicles after testosterone treatment (Rametti et al., 2012). These FA increments could be due to increases in the number of microtubules and macromolecules induced by testosterone. In summary, the effects of testosterone on the brain of FtMs should be interpreted as an anabolic side effect.

Contrary to FtMs, the suppression of testosterone in MtFs via antiandrogens would diminish the anabolic tone in brain tissues and induce decreases (Zubiaurre-Elorza et al., 2014). Moreover, the effect of estradiol administration could also play an additional role via its adverse effects on the brain (Zubiaurre-Elorza et al., 2014).

Estradiol is selectively cytotoxic to β-endorphin neurons and perhaps other cells. The mechanism involves aromatic hydroxylation of estradiol to catechol estrogens and the production of free radicals in the hypothalamus (Brawer, Beaudet, Desjardins, & Schipper, 1993); the developing human hypothalamus and cerebral cortex can produce catechol estrogens (Fishman, Naftolin, Davies, Ryan, & Petro, 1976).

Finally, morphological decreases are not only seen in MtFs after cross-sex hormone treatment since menopausal estrogen therapy has also been associated with smaller regional volumes in frontal, temporal, and limbic regions as well as the hippocampus (Casanova et al., 2011; Resnick et al., 2009). Moreover, estrogen therapy has been associated with greater brain atrophy among women aged 65 years or older (Resnick et al., 2009). Taking all these observations together, the brain decreases observed in MtFs after cross-sex hormone treatment should be interpreted as side effects due to a loss of the anabolic tone produced by suppression of testosterone combined with some deleterious effects from estradiol.

### Clinical Consequences

Do cross-sex hormone treatment induced brain changes have clinical consequences? The few existing studies on transsexuals report on short- to medium-term treatment durations and during this period cross-sex hormone treatment seems to be reasonably safe for clinical variables associated with the vascular system, metabolism, and skeleton (Gooren, 2011; Wierckx et al., 2012). However, nothing is known about older transsexuals; long-term clinical studies are yet to be published, and risks may become more apparent as the duration of hormone exposure increases (Gooren, 2011). Up until now, neuropsychological research on cross-sex hormone treatment in transsexuals has been mainly focused on the effects of gonadal hormones on sexually dimorphic emotional and cognitive behaviors (van Goozen, Cohen-Kettenis, Gooren, Frijda, & van de Poll, 1995). From a clinical viewpoint, neuropsychological tests that are sensitive to subtle brain changes and tests that are sensitive to the cognitive functions subserved by the structures that experience the most significant changes after treatment are needed and have yet to be carried out.

## Postmortem Studies of the Brain of Male-to-Female Transsexuals

For the sake of clarity, this review first discussed in vivo studies on brains of MtFs and FtMs before and after cross-sex hormone treatment. However, historically, the first studies of the transsexual brain were done in postmortem specimens of cross-sex hormone-treated MtFs. These seminal studies are better understood after reading the previous sections.

All the postmortem studies on MtFs have been done in Swaab’s laboratory in the Netherlands Institute for Neuroscience. They patiently collected up to a dozen hypothalami and adjacent regions of cross-sex hormone-treated MtFs and one FtM transsexual. With respect to sexual orientation, their MtFs are a mixture of homosexual, nonhomosexual, and unknown sexual orientation and also a mixture of early- and late-onset GD (Garcia-Falgueras & Swaab, 2008).

### Postmortem Brain Weight

The postmortem data on the weight of the brain of MtFs are not conclusive (Table 10). In a first report, MtFs had a brain weight that did not differ from that of the studied male and female control specimens (Zhou et al., 1995). When more subjects were included in the study, it was found that brain weight was almost significantly lower than those of control males, but did not differ from those of control females (Garcia-Falgueras, Ligtenberg, Kruijver, & Swaab, 2011; Garcia-Falgueras & Swaab, 2008; Table 10). As was seen above, this “in between” position for the MtF brain weight seems to be a consequence of the cross-sex hormone treatment and not a phenotypic characteristic of the MtFs; rather, it would be the dramatic effect of estradiol and antiandrogen treatment on their gray matter (Hulshoff Pol et al., 2006; Zubiaurre-Elorza et al., 2014).

**Table 10:** Brain weight in a mixed sample of treated homosexual and nonhomosexual male-to-female transsexuals

Brain region/aspect	Normative sex differences (M vs. F)	Sample size and age (range)	Sample source	Brain-relevant sample characteristics	Brain methods and measurements	Statistically controlled confounding variables	Selected findings and conclusions	Authors
Brain Weight	M > F	6 MtF (2 Hom; 3 Nonhom; 1 Bi) 9 Hom M (AIDS) 10 F (2 postmenopausal; 1 AIDS; 2 abnormal hormone levels) 15 presumed Het M (3 AIDS; 4 abnormal hormone levels)	Netherlands Institute for Neuroscience, Netherlands	MtF cross-sex hormone treated	Anatomopathology *Measurement* Postmortem brain weight	Matched age, postmortem time, duration of formalin fixation	No significant group differences for brain weight *Conclusions* brain weight of MtF do not differ from male and female controls’	Zhou et al. (1995)
Brain Weight	M > F	6 MtF (same Zhou et al. (1995) + 1 untreated) 10 Het M (3 sex hormone disorders) 9 Hom M 10 F (3 sex hormone disorders) 1 FtM	Netherlands Institute for Neuroscience, Netherlands	MtF cross-sex hormone treated	Anatomopathology *Measurement* Postmortem brain weight	Matched age, postmortem time, duration of formalin fixation	No significant group differences for brain weight *Conclusions* brain weight of MtF do not differ from males and female controls	Kruijver et al. (2000)
Brain Weight	M > F	12 MtF (7 Nonhom; 2 Hom; 3 unknown sexual orientation) 14 Het M 11 Het F Age range: MtF: 26–84 Het M: 25–81 Het F: 21–82	Netherlands Institute for Neuroscience, Netherlands	MtF cross-sex hormone treated	Anatomopathology *Measurement* Postmortem brain weight	Matched age, postmortem time, duration of formalin fixation	No significant group differences for brain weight *Conclusions* brain weight of MtF do not differ from males and female controls’	Garcia-Falgueras and Swaab (2008)

*AIDS* acquired immune deficiency syndrome, *GD* gender dysphoria, *SD* sex differences, *M* male, *F* female, *Ss* subjects, *MtF* male-to-female transsexuals, *Hom* homosexual, *Nonhom* nonhomosexual transsexuals, *Het* heterosexual, *Bi* bisexual

### Hypothalamic Nuclei and the Bed Nucleus of the Stria Terminalis

A land-mark in the study of the brain of transsexuals was the postmortem neurohistological report showing that hormonally treated MtFs have a feminine central part of the bed nucleus of the stria terminalis (BSTc) (Zhou et al., 1995). Their laboratory had previously reported sex differences in the human hypothalamus (Hofman & Swaab, 1989; Swaab & Hofman, 1988) and tried to relate some nuclei of this region with sexual orientation (Swaab, Gooren, & Hofman, 1992), a line of research followed by others (Allen & Gorski, 1990; LeVay, 1991). The interest of the Swaab group in the BST had arisen from previous animal studies showing that this olfactory nucleus, implicated in sexual behavior (Claro, Segovia, Guilamon, & Del Abril, 1995; Emery & Sachs, 1976), was sexually dimorphic in guinea pigs (Hines, Davis, Coquelin, Goy, & Gorski, 1985) and rats (del Abril et al., 1987; Guillamon, Segovia & del Abril, 1988). Moreover, at that time, it was already known that BST neurons have androgen and estrogen receptors (Simerly, Chang, Muramatsu, & Swanson, 1990) as well as aromatase activity (Jakab, Horvath, Leranth, Harada, & Naftolin, 1993). Indeed, Allen and Gorski (1990), in human postmortem material, had shown sex differences in the darkly stained posterior medial region of the BST (BSTdsmp), where males showed a larger volume than females. It should be noted that the BST and the central and medial amygdaloid nuclei constitute the extended amygdala (de Olmos et al., 1978; de Olmos & Ingram, 1972).

The studies of the Swaab group are summarized in Table 11. In humans, the volume of the BSTc, calculated after immunocytochemical staining of vasoactive intestinal peptide (VIP) or somatostatin (SOM) fibers, is greater in male than in female controls (Kruijver et al., 2000; Zhou et al., 1995). These differences become significant only in adulthood (Chung, De Vries, & Swaab, 2002). Generally, males had stronger nuclear androgen immunoreactivity than females. The male hypothalamus showed intense androgen receptor immunoreactivity, while weaker labeling was found in the BST (Fernandez-Guasti et al., 2000). In some areas of the hypothalamus, ARs are unrelated to transsexuality (Kruijver, Fernandez-Guasti, Fodor, Kraan, & Swaab, 2001). However, in MtFs, the volume and the number of SOM neurons in the BSTc are feminine (Kruijver et al., 2000; Zhou et al., 1995; Table 11).

**Table 11:** Selected findings from Swaab laboratory on postmortem brain specimen of male-to-female transsexuals

Structure and stained cells	Normative pattern of sexual dimorphism	MtFs		References
		Volume	Neurons number	
BSTC				
VIP	M > F	Feminine		Zhou et al. (1995)
Somatostatin	M > F	Feminine	Feminine	Kruijver et al. (2000)
INAH-1				
Thionin	M > F	Masculine	Masculine	
Galanin	M > F	Masculine	Masculine	
VIP	M > F	Masculine	Masculine	
INAH-3	M > F	Feminine	Feminine	García-Falgueras and Swab (2008)
Thionin	M > F	Isomorphic	Isomorphic	
NPY	M = F	Isomorphic	Isomorphic	
INAH-4	M = F			
Uncinate (ANAH-3 + ANAH-4)				
NPY	M = F	Isomorphic	Isomorphic	
Synaptophysin	M = F	Isomorphic	Isomorphic	
Infundibular nucleus	F > M	Feminine	Feminine	Taziaux et al. (2012)
NKB	F > M	Feminine	Feminine	
Kisspeptin	F > M		Feminine	Taziaux et al. (2016)
Paraventricular nucleus	M = F	Isomorphic	Isomorphic	Zhou et al. (1995)
Suprachiasmatic nucleus	M = F	Isomorphic	Isomorphic	

*BSTC* central region of the bed nucleus of stria terminalis, *INAH* interstitial nucleus of anterior hypothalamus (1, 3, 4), *VIP* vasointestinal peptide, *NPY* neuropeptide Y, *NKB* neurokinin B. Except for thionin staining, under each structure is indicated the type of cells marked using immunocytochemical techniques

Swaab’s laboratory has examined several other structures, including the interstitial nucleus of the anterior hypothalamus number 1 (INAH-1), the INAH-3, the INAH-4, and the infundibular, paraventricular, and suprachiasmatic nuclei of the hypothalamus (Table 11). The INAH-1 (Allen, Hines, Shryne, & Gorski, 1989) is also known as the sexually dimorphic nucleus of the preoptic area (SDN-POA) (Swaab & Fliers, 1985) or the intermediate nucleus of the preoptic area (InM, Garcia-Falgueras et al., 2011). Sex differences in the INAH-1 are controversial with positive (Swaab & Fliers, 1985) and negative reports (Allen et al., 1989; Byne et al., 2000; LeVay, 1991). Males have a larger INAH-1 after thionin and galanin neuron staining. However, the INAH-1 of MtFs is masculine since these subjects do not significantly differ from male controls (Garcia-Falgueras et al., 2011). This confirmed previous results on this nucleus from the same group (Zhou et al., 1995).

The paraventricular and suprachiasmatic nuclei of the hypothalamus seem to be masculine in MtFs (Zhou et al., 1995). The uncinate hypothalamic nucleus, composed of the INAH-3 and INAH-4, has also been studied. In the INAH-3, when thionin, but not neuropeptide Y (NPY), was used as a stain, males showed a larger volume and greater number of neurons than females (Garcia-Falgueras & Swaab, 2008). The volume and number of neurons in the INAH-3 of MtFs were similar to those in control females when thionin staining was used. It should be noted that in homosexual men the volume of the INAH-3 was reported to be feminine (LeVay, 1991).

Peptidergic neurons synthesizing kisspeptin (KS) and neurokinin B (NKB) in the infundibular nucleus of the hypothalamus (INF) play a role in the secretory output of gonadotropin-releasing hormone (GnRH) and show an F > M pattern of sex differences (Hrabovszky et al., 2010). Recently, it was reported that an estimation of INF volume, based on neurokinin B-cell immunoreactivity, showed larger human NKB system volumes in females than males (Taziaux, Swaab, & Bakker, 2012). NKB immunoreactivity was reported to be typically female in the InF of MtFs (Taziaux et al., 2012). Moreover, MtFs also showed a feminine pattern in the number of KS neurons in the INF (Taziaux et al., 2016).

The studies of Swaab’s laboratory on the expression of sex differences in hypothalamic nuclei and the BSTc of MtFs represent an extraordinary effort and underscore the importance of developmental processes in MtFs. The postmortem studies as a whole suggest the following.

First, is the BSTc a marker of male-to-female transsexualism? Several arguments challenge this. First, Chung et al. (2002) in their ontogenetic study of the human BSTc from fetal life onwards found that sex differences in volume and number of neurons became significant only in adulthood. Thus, the BSTc becomes sexually dimorphic long after the symptoms of transsexualism typically appear (Lawrence & Zucker, 2014). Chung et al. (2002), on the basis of the study by Davis et al. (1996) on the ontogeny of the anteroventral periventricular nucleus of the hypothalamus (AVPv), argued that organizational effects of testosterone on sexual differentiation may become clear later in life. Davis et al. (1996) found that the length of the AVPv in rats is sexually dimorphic in adulthood between days 60 and 80 after birth. As was mentioned earlier (Fig. 1d), a similar finding was reported for the rat locus coeruleus (Pinos et al., 2001). Interestingly, both the AVPv and the locus coeruleus present a morphological pattern of F > M sex differences and have similar ontogeny. However, the morphological pattern of sex differences of the BSTc is M > F and this pattern has a different type of ontogeny. The ontogeny of the M > F pattern of sex difference has been investigated in the bed nucleus of the accessory olfactory tract and differences appeared early in life (Collado, Segovia, & Guillamon, 1998; Fig. 1c); one would expect this type of pattern for the human BSTc. Moreover, testosterone promotes growth in the M > F pattern but inhibits it in the F > M pattern (Guillamon & Segovia, 1996).

Second, postmortem human studies, as in other mammals, show that sex differences appear in two morphological patterns M > F (BSTc, INAH-3) and F > M (INF), so the feminization of MtFs in these nuclei occurs because the morphological measurements either decrease (BSTc, INAH-3) or increase (INF). All the postmortem studies were done on cross-sex hormone-treated MtFs and the possible effects of the hormone treatment cannot be discounted. Feminization treatment of MtFs requires the administration of pharmacologically active doses of estradiol to natal men. The adverse effects of estradiol on the hypothalamus (Hulshoff Pol et al., 2006) and the right thalamus, pallidum, and CTh (Zubiaurre-Elorza et al., 2014) have been reported and discussed in the previous section and also noted elsewhere (Lawrence & Zucker, 2014). Moreover, the effects of testosterone suppression by antiandrogens were underscored above (Zubiaurre-Elorza et al., 2014). Therefore, it is not possible to discount deleterious effects from estradiol being the explanation for the “feminization” in the INAH-3 or the BSTc of MtFs. These nuclei present an M > F sexually dimorphic pattern. It could be the case that the morphological decreases interpreted as “feminine” in these nuclei might only reflect the negative effects of the high doses of estradiol plus antiandrogens that MtFs chronically receive (Hulshoff Pol et al., 2006; Zubiaurre-Elorza et al., 2014). The feminine INF in MtFs (Taziaux et al., 2012, 2016) is a completely different case because the pattern of sexual dimorphism in this nucleus is F > M and on this occasion the pattern of sex differences precludes a biased interpretation because the deleterious effect of the treatment predicts morphological decreases and yet the F > M pattern was still observed in this nucleus in MtFs.

Third, throughout this review we have made a point of the importance of distinguishing the sexual orientation and onset of gender dysphoria in MtFs and FtMs. With respect to sexual orientation, the postmortem studies of MtFs are a mixture of homosexual, nonhomosexual, and unknown sexual orientation and also a mixture of early and late onset of the GD (Garcia-Falgueras & Swaab, 2008). It has been suggested that the sample of these studies might be consistent with the hypothesis that all were nonhomosexual transsexuals (Lawrence & Zucker, 2014). Recently, it was noted (Lawrence & Zucker, 2014) that a voxel-based morphometry study of pedophilic offenders found reduced gray matter in the amygdale, hypothalamus (bilaterally), septal regions, substantia innominata, and the BST (Schiltz et al., 2007). Lawrence and Zucker (2014) suggested that a feminine BSTc might be “a marker for paraphilic male sexuality or for only nonhomosexual MtF transsexualism, rather than for all types of transsexualism. Alternatively, the BSTc findings may be attributable to the effects of transgender hormone therapy” (p. 621). If we take into account the in vivo studies on the brain effects of cross-sex hormone treatment, the later might be the more probable explanation.

Fourth, the postmortem studies show that the feminization of MtFs seems to affect cells that express specific peptides because feminization is detected in cells that express NKB and KS immunoreactivity (INF) and VIP (BSTc) and galanin (SDN-POA) but not NPY or synaptophysin (INAH-3). This reflects the complexities of sex differences’ expression, which types of cells are feminized in (nonhomosexual?) MtFs, and constitutes a first step and a guide for future morphological and molecular analyses.

## Conclusions

Untreated MtFs and FtMs who have an early onset of their gender dysphoria and are sexually oriented to persons of their natal sex show a distinctive brain morphology, reflecting a brain phenotype. These phenotypes are different from those of heterosexual males or females; the differences affect the right hemisphere and cortical structures underlying body perception. The genesis of these phenotypes might be caused by atypical effects of sex hormones or their metabolites in specific cortical regions of MtFs and FtMs. These effects of hormones on the cortex suggest the hypothesis that brain differences between homosexual MtFs and FtMs and male and female controls are due to differences in the development of the cortex; this hypothesis would imply that the thinning process undergone by some regions of the cortex is timed differently in each phenotype.

The review of the available data seems to support two existing hypotheses: (1) a brain-restricted intersexuality in homosexual MtFs and FtMs and (2) Blanchard’s insight on the existence of two brain phenotypes that differentiate “homosexual” and “nonhomosexual” MtFs. The studies on the effects of cross-sex hormone treatment on the brain of MtFs and FtMs consistently indicate dramatic effects on the gray and white matter after short- to medium-term treatments but the long-term effects on the brain require evaluation. Finally, the postmortem studies should be interpreted in light of these in vivo findings as well as of their underlying mechanisms.
